# Acute environmental temperature variation affects brain protein expression, anxiety and explorative behaviour in adult zebrafish

**DOI:** 10.1038/s41598-021-81804-5

**Published:** 2021-01-28

**Authors:** S. Nonnis, E. Angiulli, E. Maffioli, F. Frabetti, A. Negri, C. Cioni, E. Alleva, V. Romeo, G. Tedeschi, M. Toni

**Affiliations:** 1 Department of Veterinary Medicine, Università degli Studi di Milano, Via dell’Università 6, 26900 Lodi, Italy; 2 CRC “Innovation for Well-Beeing and Environment” (I-WE), Università degli Studi di Milano, Milano, Italy; 3 Department of Biology and Biotechnology ‘‘Charles Darwin”, Sapienza University, Via Alfonso Borelli 50, 00161 Rome, Italy; 4 CIMAINA, Università degli Studi di Milano, Milano, Italy; 5 Department of Experimental, Diagnostic and Specialty Medicine, University of Bologna, Bologna, Italy; 6 Center for Behavioural Sciences and Mental Health, IstitutoSuperiore di Sanità, Rome, Italy

**Keywords:** Proteomics, Behavioural methods, Proteomic analysis

## Abstract

This study investigated the effect of 4-d acute thermal treatments at 18 °C, 26 °C (control) and 34 °C on the nervous system of adult zebrafish (*Danio rerio*) using a multidisciplinary approach based on behavioural tests and brain proteomic analysis. The behavioural variations induced by thermal treatment were investigated using five different tests, the novel tank diving, light and dark preference, social preference, mirror biting, and Y-Maze tests, which are standard paradigms specifically tailored for zebrafish to assess their anxiety-like behaviour, boldness, social preference, aggressiveness, and explorative behaviour, respectively. Proteomic data revealed that several proteins involved in energy metabolism, messenger RNA translation, protein synthesis, folding and degradation, cytoskeleton organisation and synaptic vesiculation are regulated differently at extreme temperatures. The results showed that anxiety-like behaviours increase in zebrafish at 18 °C compared to those at 26 °C or 34 °C, whereas anxiety-related protein signalling pathways are downregulated. Moreover, treatments at both 18 °C and 34 °C affect the exploratory behaviour that appears not to be modulated by past experiences, suggesting the impairment of fish cognitive abilities. This study is the continuation of our previous work on the effect of 21-d chronic treatment at the same constant temperature level and will enable the comparison of acute and chronic treatment effects on the nervous system function in adult zebrafish.

## Introduction

Temperature is one of the most important factors influencing fish physiology and behaviour^1^ because poikilothermic animals depend on the environmental temperature to regulate their body temperature. Each species lives within a given temperature range, which is the result of the evolution and adaptation to its environmental niche; this range can be represented graphically by the temperature tolerance polygon^2^.

Significant perturbations of the environmental temperatures are perceived by fish as stressful events and induce both behavioural and physiological adjustments to maintain their homeostatic balance. The behavioural response comprises migration to areas with a more favourable temperature^3^, while the physiological response is often the result of a complex series of cellular, tissue, and organismal events, defined as acclimatisation, that allow the individual fish to modify its thermal sensitivity^4^. Therefore, temperature variations may influence the growth rate, food consumption, feed conversion, physiology, behaviour, and other body functions^5–10^.

Climate change in recent decades has increased the attention on the impact of thermal variations on the aquatic environment, and several studies have investigated the effect of temperature on freshwater and saltwater fish species^11–27^. The influence of thermal perturbations depends on the extent of the variation and its duration so that short (acute) or long (chronic) exposure to the same temperature may induce different responses. Generally, acute exposures induce thermal stress while chronic exposures may result in compensatory adjustment due to acclimatisation^28^.

The definitions of “acute” and “chronic” terms are not clearly stated. Acute stress usually refers to a stimulus that lasts from minutes to days, whereas chronic stress is a stimulus that persists from days to months, depending on the study^29–32^. Catching fish with nets and handling animals on farms are often considered acute stress^33,34^, while fish overcrowding in the intensive farms and the consequent water pollution are considered chronic stress^34–36^. According to Logan et al.’s review on the transcriptomic responses to environmental temperature^22^, in the present paper, we considered a temperature exposure lasting less than 5 d as acute and a more prolonged exposure as chronic.

In this paper, we have studied the effect of acute thermal treatments at 18 °C, 26 °C (control) and 34 °C on the brain proteome and behaviour of zebrafish, a poikilotherm and eurytherm cyprinid increasingly used in recent years in many areas of scientific research. Given its thermal tolerance, zebrafish represents a good model to study the effects of thermal variation in terms of behavioural and biochemical modifications. Indeed, behavioural tests have been successfully performed in zebrafish^37–43^, and the availability of its genome has allowed specific transcriptomic^44–49^ and proteomic analyses^14,50^.

Zebrafish live in India, mainly near the Ganges River^51^, where there are daily temperature fluctuations of approximately 5 °C and wide seasonal temperature variations ranging from 6 °C in the winter to more than 38 °C in the summer^52^. Zebrafish are characterised by wide thermal tolerance from 6.7 to 41.7 °C^53,54^, enabling us to test the effects of large temperature variations within the animal's tolerance polygon^55^.

Previous research from our group investigated the effect of chronic exposure (21 d) to the same temperatures of 18 °C, 26 °C and 34 °C on adult zebrafish^14,56^. The three temperature values were chosen according to Vergauwen et al.^49^ within the zebrafish vital range and correspond to temperatures the fish cope with in the natural environment. Our results showed that adult zebrafish at 18 °C and 34 °C have altered expression of proteins associated with metabolism, cytoskeleton organisation and cellular transport. This suggests that environmental temperature strongly impacts the cytoarchitecture and energy state of the brain^14^. In particular, we found that reduced expression of synaptic proteins and downregulated protein pathways were positively correlated with impairments of the cognitive abilities of fish in the Y-Maze test. In addition, behavioural tests showed that long exposure to 34 °C reduces the anxiety-like status of zebrafish^56^.

We aimed to further investigate the effect of temperature on the zebrafish nervous system by analysing the brain proteome and behaviour of adult specimens subjected to 4-d acute thermal stress at the same temperatures, 18 °C, 26 °C (control) and 34 °C. Behaviour was tested using the same experimental design—i.e., novel tank diving (NTT), light and dark preference (LDT), social preference (SPT), mirror biting (MBT) and Y-Maze (YMT). The results enabled us to compare the effect of the 4-d acute thermal treatment to the 21-d chronic one previously studied^14^.

## Methods

### Ethical note

Animal experiments were performed in accordance with the guidelines approved by the Animal Care Committee and authorized by the Italian Ministry of Health (protocol number 290/2017-PR), and the animals were handled in accordance with the European directive 2010/63 on the protection of animals used for scientific purposes. The health status and the well-being of all animals involved in the study were checked daily for the duration of the thermal treatment and the subsequent behavioural tests. No procedures caused significant pain or lasting harm to the zebrafish, and no experimental subject died during the experimental procedures (fish housing, heat treatment and behavioural tests).

### Subjects

A total of 108 experimentally-naïve adult (12 months old) wild type zebrafish (1:1 male: female sex ratio) from a stock colony purchased from commercial dealers acclimated at 26 ± 1 °C in the zebrafish facility of the University of Bologna, was used in the present study. Fish were randomly divided into six identical tanks (width (W) 40 × depth (D) 30 × height (H) 30 cm, home-tanks). Eighteen individuals per home-tank were maintained at the density of 1 zebrafish/l at 26 ± 1 °C (control temperature) for 10 days to acclimate to the tank (adaptation period).

### Thermal treatment

Of the six tanks, two were randomly chosen to house fish at 18 °C (home-tanks 1 and 5), two at 26 °C (home-tanks 4 and 6) and the other two at 34 °C (home-tanks 2 and 3). Water temperature was gradually (0.5 °C/h) decreased from 26 to 18 °C and raised from 26 to 34 °C to start thermal treatment. Zebrafish were then maintained at 18 ± 1 °C and 34 ± 1 °C for 4 days while control fish were maintained at 26 ± 1 °C. At the end of thermal treatment, 15 individuals from each home-tank were subjected to behavioural tests and 3 were individually euthanized by a prolonged immersion in a solution of the anaesthetic tricaine methanesulfonate MS-222 (300 mg/l), decapitated, and their brains were dissected for the proteomic analysis.

### Fish husbandry during adaptation and thermal treatment

Zebrafish husbandry was based on the same protocol used in our previous works and all procedures were conducted in the same laboratory room by the same operators using the same tanks and housing conditions previously described^14,56^. Briefly, fishes were manipulated equally in a 14/10 h light/dark photoperiod (light 6 am-8 pm). The interior enrichment of each home-tank (consisting of a heating coil, inlet and outlet pipes for the filters and an aerator) was replicated exactly. The water temperature was held constant by external water chiller (TK 150 Teco) and digital thermostats (Eden 430) connected to a heating coil (Eden 415, 230 V, 50/60 Hz, 80 W) and checked daily using an analogue thermometer. In each tank, a constant flow of filtered water (600 l/h) was maintained by an external filtration system (Eden 511 h), and the water was also continuously aerated (7.20 mgO_2_/l) by an aquarium aerator (SicceAIRlight, 3300 cc/min 200 l/h). The conductivity of the water ranged between 400 and 500 micro-Siemens. The main chemical/physical characteristics of the tank water such as total ammonia (NH_3_/NH_4_), nitrite (NO_2_), water pH, total hardness, carbonate hardness (dKH), and phosphate (PO_4_), copper (Cu) and chlorine (Cl_2_) levels were checked at least two times per week using a Sera Aqua-test Box Kit (Sera, Italy) and an eSHa Aqua Quick Test (Nayeco, Spain). No differences were found between the treatment and control tanks. The zebrafish were fed three times a day (10 am, 2 pm and 6 pm) with a commercial dry granular food (TropiGranMIX, Dajanapet). Food (0.6 g/day) was administered to each tank, allowing zebrafish at the three temperatures to feed themselves according to their appetite. The average mass (± SEM) at end of the thermal treatment is reported in Table S1. A two-way ANOVA (3 × 6, temperature/home-tank) analysis detected no statistically significant differences among masses (*P* > 0.44). During adaptation and thermal treatment, the behaviour of fish in each tank was observed every day for 5 min by two operators (M.T. and E.A.) to evaluate physical indicators for fish welfare^57^. During the four days of thermal treatment no freezing events were observed in the tanks at the three temperatures.

### Behavioural tests: general design

Fish were subjected to five behaviour tests: NTT, LDT, SPT, MBT and YMT. These are paradigms for assessing different aspects of behaviour such as the anxiety-like state, boldness, social preference and aggressive behaviour, spatial orientation and interest in novelties.

Behavioural tests were conducted using the same apparatus and protocol used in our previous studies to which reference is made for details (YMT^14^ and NTT, LDT, SPT, MBT^56^).

To reduce stress on individuals and to allow comparisons with behavioural results previously obtained after a 21-day chronic thermal treatment^14,56^, we decided to use 15 individuals exclusively for the YMT test and 15 individuals for LDT, NTT, SPT and MBT. The number of experimental subjects was chosen, given the reduction criteria, on the basis of data in the literature suggesting that in zebrafish, significant data may be obtained with n = 12–15 per group for strong effects^40^. To minimize the interference between the different tests^58,59^ the NTT and LDT were performed alternately as the first or second test, whereas the SPT and MBT were performed alternately as the third or fourth test.

The temperature of the water in all the tanks used in the behavioural tests was the same as the home-tank in which the animal had been housed and was held constant with 1 °C of maximum variation between the beginning and the end of each test. The tanks were not aerated during testing to avoid disturbing the animals. Before the tests, each single fish was captured using a beaker and placed in a waiting tank (W15 × D10 × H10 cm) for 30 min until the beginning of the behavioural tests. After each test the water was removed and the apparatus rinsed and filled with clean water. In the room, a diffuse light was present to avoid directional lighting that could interfere with fish behaviour. The duration of the NTT, LDT, SPT, MBT and of a single YMT trial was 10 min each. All tests were video recorded by a webcam (Logitech C170) placed one meter above (LDT, Y-Maze) or in front of (NTT, SPT and MBT) each apparatus. Tests were carried out in May 2018 between 10 am and 5 pm.

The parameters recorded depend on the test. The main parameters analysed relating to locomotor behaviour were the number and duration of immobile phases (sec), distance travelled (m), average and maximum speed (m/sec), total number of rotations, ratio between clockwise (CW) and counter-clockwise (CCW) rotations, absolute turn angle (the sum of the absolute angles between each movement vector of the animal, in degrees), meandering (the change in direction of movement relative to the distance moved, whose value is the result of the absolute turn angle divided by the total distance travelled in deg/m). For other parameters, see Methods and Figures relating to the specific tests.

### Novel tank diving test (NTT)

**Figure 1:**
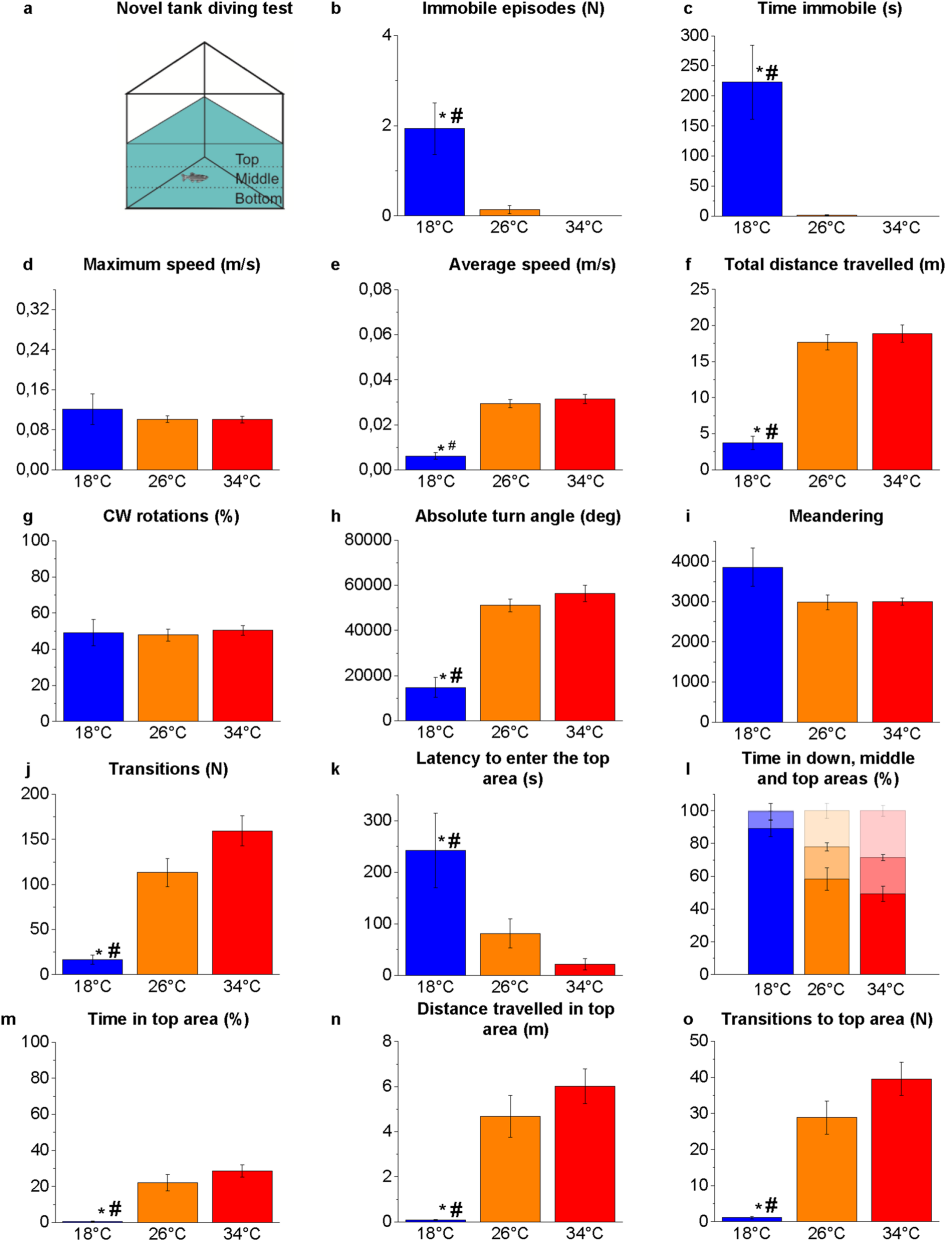
Novel environment exploration behaviour in the NTT. Schematic representation outlining the experimental design (**a**). Immobile episodes (**b**), time immobile (**c**), maximum speed (**d**), average speed (**e**), total distance travelled (**f**), clockwise (CW) rotations expressed as the percentage of total rotations (**g**), absolute turn angle (**h**), meandering (**i**), transitions among areas (**j**), latency to enter the top area (**k**), time spent in the three vertical virtual areas of the tank (**l**), time spent in the top area expressed as a percentage of the total time (**m**), distance travelled in the top area (**n**) and number of transitions to the top area (**o**). The data are expressed as means ± S.E.M. and analysed by one-way ANOVA with Bonferroni post hoc correction. *P* ≤ 0.05, * 18 °C versus 26 °C; # 18 °C versus 34 °C and $ 34 °C versus 26 °C. *P* values are reported in Table S2. N = 15.

NTT was used to evaluate the locomotor activity and anxiety-like behaviour (Fig. 1). The apparatus consisted of a glass transparent tank with a lower triangular base^60^, with sides of 30 cm and filled with 4.7 L of water. The sides of the tank were covered by white paper to avoid reflection. To measure the vertical exploratory activity, the tank was virtually divided into three equally horizontal areas (bottom, middle and top) as shown in Fig. 1a. Specific parameters recorded were latency to enter the top area (sec), time spent in the bottom/middle/top area (sec), the number of top entries and the distance travelled within the top area (m).

### Light and dark preference test (LDT)

**Figure 2:**
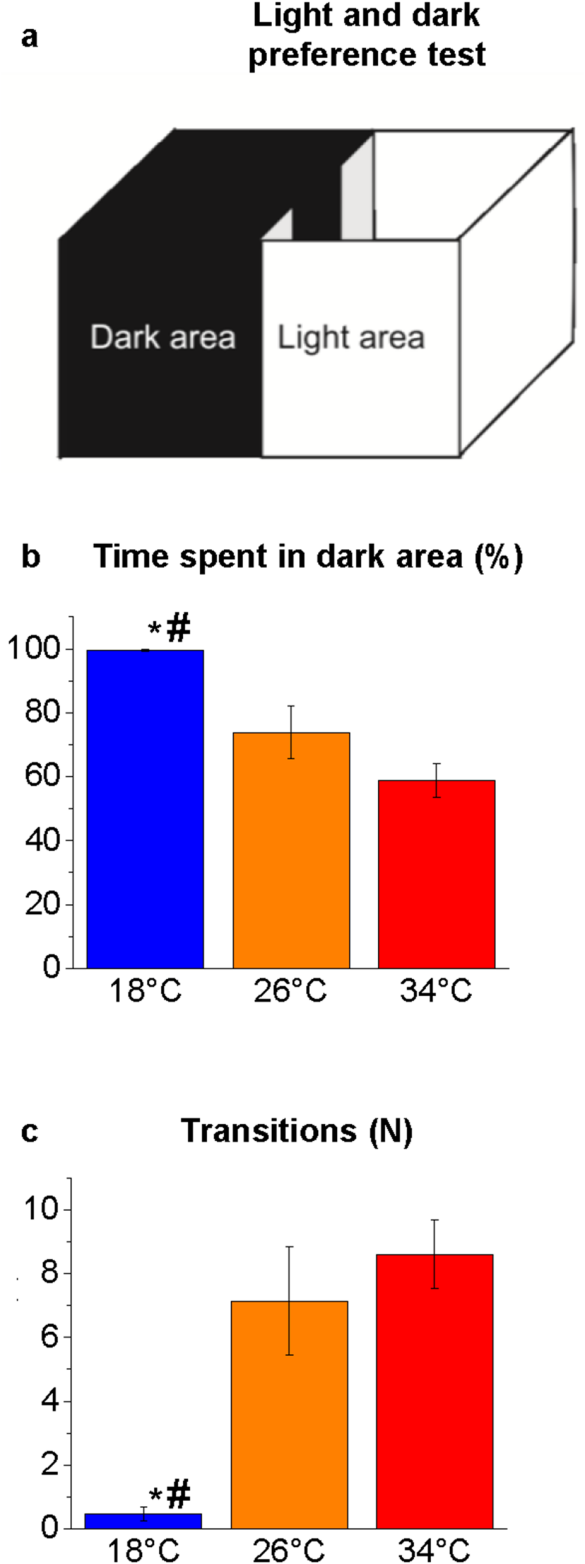
Scototaxis behaviour in the LDT. Schematic representation of the apparatus (**a**). Time spent in the dark area (**b**) and number of passages between the bright and dark areas (**c**). The data are expressed as mean ± S.E.M. and analysed by one-way ANOVA with Bonferroni post hoc correction. *P* ≤ 0.05, * 18 °C versus 26 °C; # 18 °C versus 34 °C and $ 34 °C versus 26 °C. *P* values are reported in Table S3. N = 15.

LDT was used to assess fish exploratory activity and anxiety-like behaviour (Fig. 2). This test measures the natural preference of adult zebrafish for dark environment (scototaxis) and its aversion to bright areas^61^. LDT was performed in a rectangular plastic tank (L33 × W18 × H18 cm) divided into two compartments of equal size (Fig. 2a). The lateral walls and the base of one half of the apparatus consisted of black plastic, while the other half of opaque white plastic. The dark side was shielded from ambient light with an opaque black lid^62^. The tank was filled with 4 L of water. In the middle of the tank two transparent transverse septa restrict the passage between the two areas (6 cm) to prevent that fish could freely swim from one area to the other. Two transparent sliding doors (W18 × H18 cm) define a central area (W7 × D18 × H18 cm) half black and half white where the fish was housed before starting the test. After 30 s from the insertion of the fish in the central area, the two sliding doors were lifted simultaneously to allow fish moving between black and white areas. Parameters analysed were the time spent in the bright area (sec) and the number of passages between the two areas.

### Social preference test (SPT)

**Figure 3:**
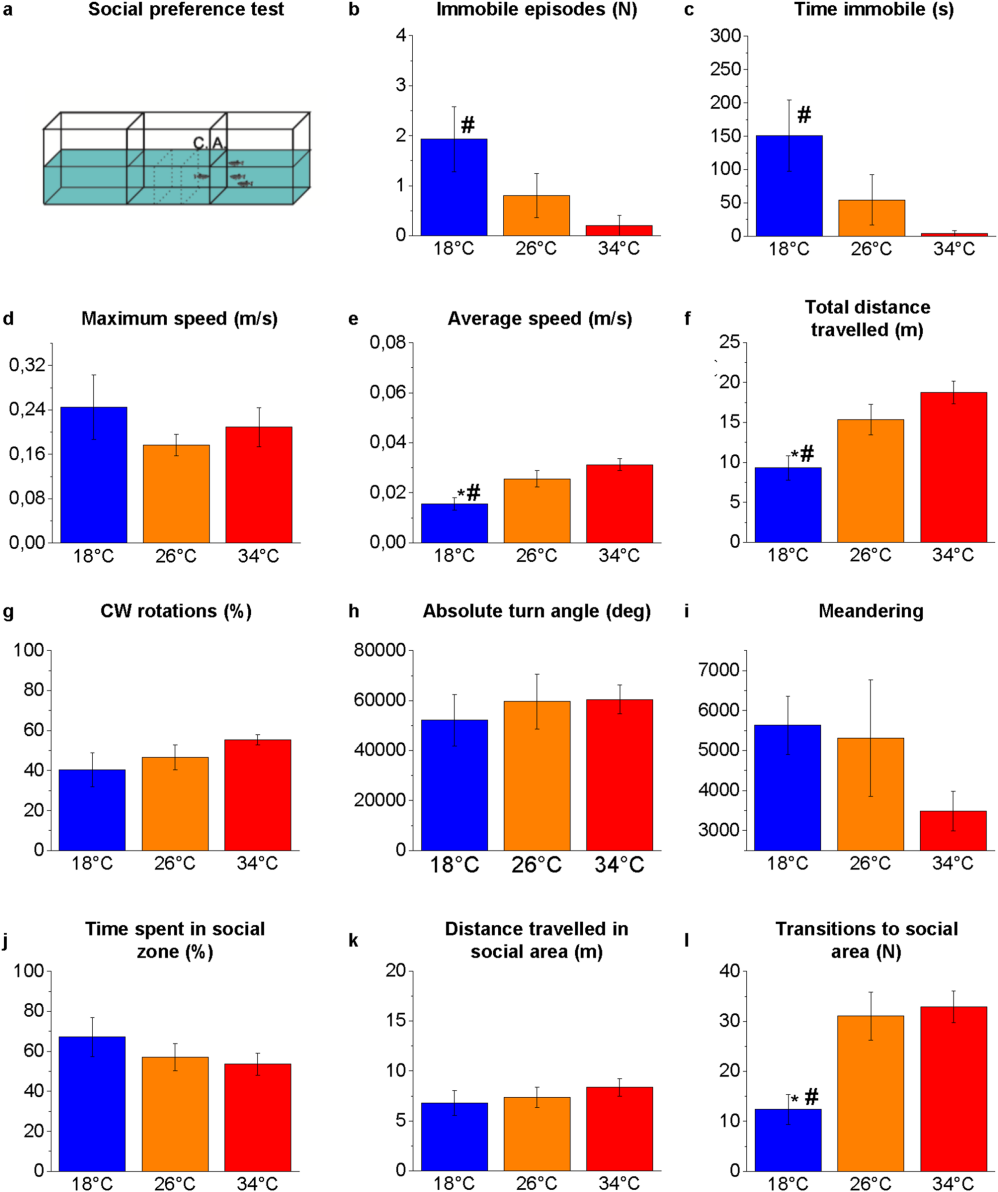
Shoaling behaviour in the SPT. Schematic representation of the apparatus (**a**). Whole tank analysis: immobile episodes (**b**), time immobile (**c**), maximum speed (**d**), average speed (**e**), total distance travelled (**f**), clockwise (CW) rotations (**g**), absolute turn angle (**h**), and meandering (**i**). Conspecific area (C.A.) analysis: time spent (**j**), total distance travelled (**k**), number of entries (**l**). The data are expressed as mean ± S.E.M. and analysed by one-way ANOVA with Bonferroni post hoc correction. *P* ≤ 0.05, * 18 °C versus 26 °C; # 18 °C versus 34 °C and $ 34 °C versus 26 °C. *P* values are reported in Table S4. N = 15.

Zebrafish are social vertebrates^63^ and exhibit group behaviours like shoaling and schooling^64,65^. The experimental setting in this test consisted of three rectangular tank (W28 × D25 × H16 cm) namely empty, experimental and conspecific tank aligned side by side in a horizontal line (Fig. 3a). Each tank was filled with 4 L of water. The experimental tank is located in the middle and houses the experimental subject. One of the two adjacent tanks (conspecific tank) contained three zebrafish representing the social stimulus, the other tank was left empty.

Three fishes of the same size and age of the experimental subjects available in the facility were used as social stimulus. The three fish were novel to the experimental subjects, having never been placed in the same tank before. The week before the tests, the three fish were transferred to the conspecific tank for 3 h a day to adapt to the tank. The same three fish were used for all social tests. The group position (on the left or the right of the experimental tank) was balanced between tests.

At the beginning of the test, two black panels were positioned between the tanks, preventing the experimental subject from seeing the other tanks. The fish was placed in the tank and left to settle for 30 s, then the two panels were gently removed. The experimental tank was divided into three virtual areas. The one closest to the conspecific tank was designated as “social area”, where fish is supposed to prefer visual interaction with conspecifics. Specific parameters analysed were the time spent in the social area (sec), distance travelled in the social area (m), and number of entries into the social area (sec).

### Mirror biting test (MBT)

**Figure 4:**
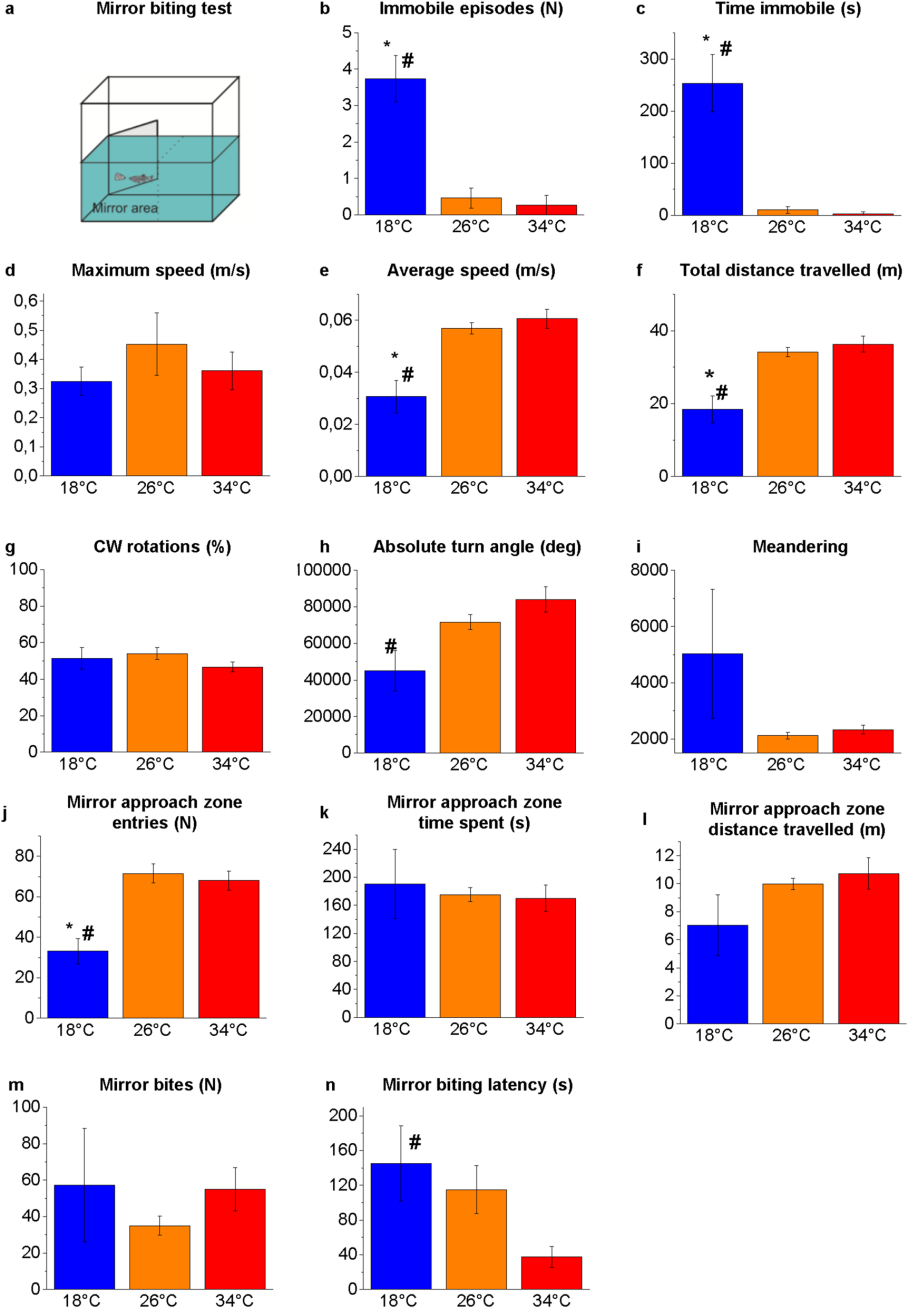
Aggressive behaviour in the MBT. Schematic representation of the apparatus (**a**). Whole-tank analysis: immobile episodes (**b**), time immobile (**c**), maximum speed (**d**), average speed (**e**), total distance travelled (**f**), clockwise (CW) rotations (**g**), absolute turn angle (**h**), and meandering (**i**). Mirror approach area: entries (**j**), time (**k**), total distance travelled (**l**), mirror bites (**m**) and mirror-biting latency (**n**). The data are expressed as mean ± S.E.M. and analysed by one-way ANOVA with Bonferroni post hoc correction. *P* ≤ 0.05, * 18 °C versus 26 °C; # 18 °C versus 34 °C and $ 34 °C versus 26 °C. *P* values are reported in Table S5. N = 15.

The mirror image stimulation is a paradigm used for studying social and aggressive behaviour in zebrafish^66^. The apparatus consisted of a barrel tank (W28 × D25 × H16 cm) filled with 4 L of water and equipped with a mirror (D16 × H14 cm) (Fig. 4a). The fish was placed in the tank and left to settle for 30 s, then the mirror was tilted at an angle of 22.5° on the long side of the tank^42,58,67^. The position of the mirror (reflected image closer to the left or right side of the aquarium) was balanced between the tests. Parameters analysed were the time spent (sec) and distance travelled (m) in the mirror area, the number of entries into the mirror area, number of times that the fish bites the mirror and the latency to the first mirror bite (sec).

### Y-Maze test (YMT)

**Figure 5:**
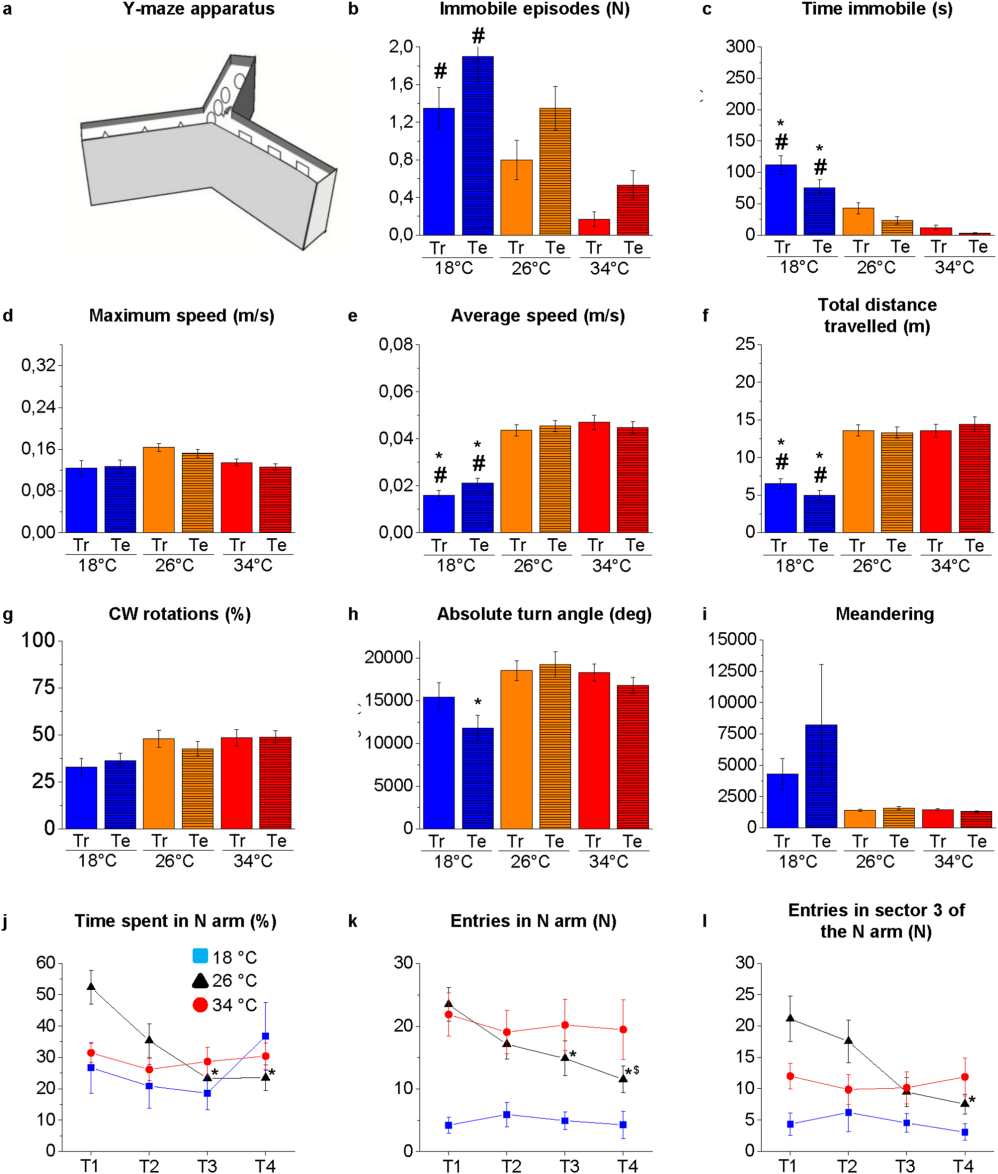
Behavioural response to thermal treatment on the Y-Maze test observed in the training (Tr) and testing (Te) phases. Schematic representation of the apparatus (**a**). Swimming activity and behaviour measured as immobile episodes (**b**), time immobile (**c**) maximum speed (**d**), average speed (**e**), total distance travelled (**f**), clockwise (CW) rotations expressed as percentage of total rotations (**g**), absolute turn angle (**h**), and meandering (**i**). The data were analysed by two-way ANOVA with post hoc Bonferroni correction. *P* ≤ 0.05, °, intra-temperature difference between Tr and Te; *, intra-phase differences between 18 and 26 °C; #, intra-phase differences between 18 °C and 34 °C; @ intra-phase differences between 34 °C and 26 °C. *P* values are reported in Table S6. Novel environment exploration T1–T4. Time spent in N arm (**j**), number of entries in N arm (**k**) and number of entries in sector 3 of the N arm (**l**). The data were analysed by two-way repeated measures ANOVA with post hoc Bonferroni correction. *P* ≤ 0.05, *, compared to T1,$, compared to T2. *P* values are reported in Table S7. Square, triangle and circle indicate 18 °C, 26 °C, and 34 °C respectively. N = 15.

The Y-Maze was composed by three arms at 120 degrees to each other (W 25 × D 8 × H 15 cm) replicating exactly the same features of the maze successfully used on zebrafish in previous works^14,41^ (Fig. 5). A single arm was identified by the presence of geometric shapes (square, circle and triangle) that do not induce fear in the fish and for which the fish has a similar preference, as demonstrated by Cognato and collaborators^41^.

The maze was filled with 4 L of water and the water depth was 6.5 cm, enough to submerge the geometric shapes. One single task consisted of four trials (T1, T2, T3 and T4) separated by a one-hour interval. Each trial consisted of a training phase (Tr) in which the fish was free to swim in the start (S) arm and in the other (O) arm for 5 min but it could not access the novel (N) arm for the presence of a dividing wall, and of a testing phase (Te) in which the wall was removed and the fish was free to swim for 5 min all over the maze also exploring the novel environment constituted by the N arm. The assignment of circle, square, and triangle to the S, O and N arm was randomized for each experimental subject. The interest in the new environment was evaluated in the testing phase by quantifying the time spent and the number of entries in N arm. To determine whether the temperature influences fish tendency to explore the novel environment in its entirety, the N arm was virtually divided into three equal parts named sector 1, 2 and 3 starting from the centre of the maze and the number of entries in the sector 3 was analysed.

### Video tracking and statistical analysis

Videos were analysed using the ANY-Maze® software (Stoelting Co., Wood Dale, IL, USA). Results were expressed as means ± SEM. Data were subjected to ANOVA analysis with a post hoc test utilising Bonferroni correction (for more detail refer to the relative figure caption). Differences were considered to be statistically significant at *P* ≤ 0.05.

### Proteomic analysis

The brain proteome was analysed by a shotgun label free proteomic approach to identify and quantify expressed proteins. Six whole brains for each temperature were homogenized according to^14^. Prior to proteolysis, proteins were reducted with 13 mM DTE (30 min at 55 °C) and alkylated with 26 mM iodoacetamide (IAA; 30 min at RT). Protein digestion was performed using sequence-grade trypsin (Roche) overnight at 37 °C using a protein:trypsin ratio of 20:1. The proteolytic digest were desalted using Zip-Tip C18 before Mass Spectrometric (MS) analysis^68^.

LC–ESI–MS/MS analysis was performed on a Dionex UltiMate 3000 HPLC System with a PicoFritProteoPrep C18 column (200 mm, internal diameter of 75 μm). Gradient: 1% ACN in 0.1% formic acid for 10 min, 1–4% ACN in 0.1% formic acid for 6 min, 4–30% ACN in 0.1% formic acid for 147 min and 30–50% ACN in 0.1% formic for 3 min at a flow rate of 0.3 μl/min. The eluate was electrosprayed into an LTQ Orbitrap Velos (Thermo Fisher Scientific, Bremen, Germany) through a Proxeon nanoelectrospray ion source. The MS was operated in positive mode in data-dependent acquisition mode to automatically alternate between a full scan (m/z 350–2000) in the Orbitrap (at resolution 60,000, AGC target 1,000,000) and subsequent CID MS/MS in the linear ion trap of the 20 most intense peaks from full scan (normalized collision energy of 35%, 10 ms activation). Data acquisition was controlled by Xcalibur 2.0 and Tune 2.4 software (Thermo Fisher Scientific)^69^.

### Statistical and bioinformatic data analysis

Database search was conducted against the zebrafish Uniprot sequence database (release December 2018) with MaxQuant (version 1.6.0.4) software using the following parameters: initial maximum allowed mass deviation of 15 ppm for monoisotopic precursor ions and 0.5 Da for MS/MS peaks, trypsin enzyme specificity, a maximum of 2 missed cleavages, carbamidomethyl cysteine as fixed modification, N-terminal acetylation, methionine oxidation, asparagine/glutamine deamidation and serine/threonine/tyrosine phosphorylation as variable modifications. Quantification was performed using the built in XIC-based label-free quantification (LFQ) algorithm using fast LFQ^70^. False protein identifications (5%) were estimated by searching MS/MS spectra against the corresponding reversed-sequence (decoy) database. Statistical analysis was performed using the Perseus software (version 1.5.5.3, www.biochem.mpg.de/mann/tools). Four technical replicates were carried out for each treated group (18 °C and 34 °C) and for 26 °C used as the control. Only proteins present and quantified in 75% of the repeats were positively identified and used for statistical analysis. An ANOVA test (cut-off at 0.01 FDR) was carried out to identify proteins differentially expressed among the three conditions^71^.

To find relevant patterns and specific differences in processes related to the diverse conditions (18 °C, 34 °C, or 26 °C) a principal component analysis (PCA) was carried out on the total brain proteome of zebrafish. The analysis was applied to all the proteins found in these three experimental conditions.

Focusing on specific comparisons, 18 °C versus 26 °C and 34 °C versus 26 °C, proteins were considered differentially expressed if they were present only in one condition or showed significant t-test difference (Welch's test *P* ≤ 0.05)^72^. Bioinformatic analyses were carried out by Panther software (Version 10.0), DAVID software (release 6.7) and ClueGO to cluster enriched annotation groups of Molecular Function, Biological Processes, and Pathways within the set of identified proteins. Functional grouping was based on *P* ≤ 0.05 and at least three counts. Bioinformatic analyses of results obtained in chronic conditions have been performed using data published in^14^.

The mass spectrometry proteomics data have been deposited to the ProteomeXchange Consortium via the PRIDE^73^ partner repository with the dataset identifier PXD016847.

## Results and discussion

### Behavioural analysis

Our previous studies demonstrated that the exposure of adult zebrafish at three different temperatures (18 °C, 26 °C or 34 °C) for 21 d affects the brain proteome and fish behaviour^14,56^. In this work, fish were exposed to the same temperatures for a shorter time (4 d) to distinguish between variations in the brain proteome and behaviour following acute and chronic thermal treatment. The effect of the exposure to the three temperatures for 4 d was assessed by behavioural tests already successfully used in adult zebrafish, such as the NTT, LDT, SPT, MBT and YMT.

### Locomotor and swimming activity

The locomotor and swimming activities of zebrafish at the three temperatures were analysed by a webcam placed in front of the tanks in the NTT (Fig. 1), SPT (Fig. 3), MBT (Fig. 4) and above the maze in the YMT (both in Tr and Te) (Fig. 5). The results showed some statistically significant differences at 18 °C versus 26 °C and 18 °C versus 34 °C, but no significant differences were detected between 34 and 26 °C (Figs. 1, 3–5 and Tables S2, S4–S6).

An increase in the time of immobility (freezing) was observed at 18 °C versus 26 °C and 18 °C versus 34 °C in the NTT (*P* = 0.0002 and *P* = 0.0002, respectively; Fig. 1c), MBT (*P* < 0.0001 and *P* < 0.0001, respectively; Fig. 4c), and YMT Tr (*P* = 0.0014 and *P* < 0.0001, respectively; Fig. 5c) and Te (*P* < 0.0001 and *P* < 0.0001, respectively; Fig. 5c), was associated with an increase in immobile episodes in the NTT (*P* = 0.0014 and *P* = 0.0006, respectively; Fig. 1b) and MBT (*P* < 0.0001 and *P* < 0.0001, respectively; Fig. 4b).

The observation by the operators of the experimental subjects in the home-tanks did not detect immobile episodes at the three temperatures during the 4 d of thermal treatment. Thus, the freezing events observed in the behavioural tests at 18 °C were not simply induced by low temperature but may represent a behavioural response to the challenge of the test in zebrafish kept at that temperature. Because freezing events are associated with anxiety^74^, it is conceivable that the challenge of the behavioural test increased more the anxiety-like status in zebrafish at 18 °C than in those at 26 °C and 34 °C. After all, behavioural tests were carried out by single individuals that could not take refuge in the school of conspecifics; this situation could have increased the animal’s anxiety. Numerous studies agree that the main function of shoaling is predator avoidance and that an increase in anxiety is associated with an increase in shoaling^37,75–78^. However, in the SPT, the only test in which the experimental subject could see other conspecifics, no significant increase was found in the number and duration of freezing events in subjects at 18 °C compared with the control (26 °C) (*P* = 0.2817 and *P* = 0.2356, respectively; Fig. 3b,c). Further experiments based on shoaling tests^65,66,79,80^ that are more suitable for assessing the animal's tendency to aggregate into groups are needed to verify whether acute or chronic thermal treatment affects shoaling and schooling behavior in zebrafish.

A reduction in the average speed was observed at 18 °C versus 26 °C and 18 °C versus 34 °C in the NTT (*P* < 0.0001 and *P* < 0.0001, respectively; Fig. 1e), SPT (*P* = 0.0417 and *P* = 0.0007, respectively; Fig. 3e), MBT (*P* = 0.0004 and *P* = 0.0001, respectively; Fig. 4e) and YMT Tr (*P* < 0.0001 and *P* < 0.0001, respectively; Fig. 5f) and Te (*P* < 0.0001 and *P* < 0.0001, respectively; Fig. 5e). Moreover, a reduction in distance travelled was observed at 18 °C versus 26 °C and 18 °C versus 34 °C in the NTT (*P* < 0.0001 and *P* < 0.0001, respectively; Fig. 1f), SPT (*P* = 0.0389 and *P* = 0.0006, respectively; Fig. 3f), MBT (*P* = 0.0004 and *P* = 0.0001, respectively; Fig. 4f) and YMT Tr (*P* < 0.0001 and *P* < 0.0001, respectively; Fig. 5f) and Te (*P* < 0.0001 and *P* < 0.0001, respectively; Fig. 5f). By contrast, no difference was observed in the maximum speed among the heat treatments (Figs. 1, 3–5 d), suggesting that exposure to different temperatures does not affect the swimming capacity of the animals.

No temperature-dependent alterations were observed in the percentage of CW and CCW rotations (Figs. 1, 3–5g) and in the meandering (Figs. 1, 3–5i) in all the tests.

### Novel tank diving test

NTT was applied to analyse the vertical exploration of the specimens in a new environment (Fig. 1a). Vertical exploration was reduced at 18 °C versus 26 °C and 18 °C versus 34 °C, as indicated by higher latency to enter the top area (*P* = 0.0120 and *P* = 0.0006, respectively; Fig. 1k) and lower values in the time spent (*P* = 0.0001 and *P* < 0.0001, respectively; Fig. 1l, m), distance travelled (*P* = 0.0001 and *P* < 0.0001, respectively; Fig. 1n) and number of transitions carried out by fish in the top area (*P* < 0.0001 and *P* < 0.0001, respectively; Fig. 1o). These results indicate greater anxiety-like status in zebrafish at 18 °C than in controls at 26 °C because the exploration of the most superficial areas in the NTT corresponds to greater boldness of the animal. By contrast, dwelling on the bottom of the tank represents an anxiety-like behaviour^38,62,74,81^.

No significant difference was observed in the comparison at 34 °C versus 26 °C, although the average values for the time spent (Fig. 1m), distance travelled (Fig. 1n) and transitions in the upper zone (Fig. 1o) were greater at 34 °C than those in the control. The same values were found to be significantly different after 21-d chronic thermal treatment^56^, indicating that exposure to high temperatures must be prolonged for several days before producing the significant alterations in the vertical exploration of the water column.

### Light and dark preference test

Behavioural analysis conducted by LDT (Fig. 2a) showed significant difference in the comparisons at 18 °C versus 26 °C and 18 °C versus 34 °C in the time spent in the dark area (*P* = 0.0079 and *P* < 0.0001, respectively; Fig. 2b) and in the number of passages between areas (*P* = 0.0007 and *P* < 0.0001, respectively; Fig. 2c). Zebrafish at 18 °C showed a total preference for the dark area where fish spend almost all the time (99.4% of the total time) compared with fish at 26 °C (73.8%) and 34 °C (58.9%) (Fig. 2b). Moreover, fish at 18 °C strongly reduced the number of crossings between the two compartments of the apparatus (Fig. 2c), confirming the preferential choice by the animal for the dark and covered region^60,61^. The results of the LDT support a major anxiety-like status in subjects at 18 °C versus 26 °C, as already found in the NTT, given that the opposite—i.e., increased exploration of the bright compartment in the LDT—is considered the expression of major animal boldness^60,61^ due to the decrease in its anxiety.

The comparison of 34 °C versus 26 °C showed a non-significant reduction in the time spent in the dark area after 4-d acute thermal treatment, and this trend was confirmed after 21-d acute thermal treatment, when the reduction was statistically significant^56^. This finding confirms that the high temperature alters the light/dark preference of adult zebrafish but an exposure longer than 4 d is required to significantly alter the phototaxis behaviour.

### Social preference test

In the SPT (Fig. 3a), no differences were detected in the time spent (Fig. 3j) and distance travelled (Fig. 3k) in the social zone among the three temperatures. However, the lower number of transitions to the social area observed in the comparisons of 18 °C versus 26 °C and 18 °C versus 34 °C (*P* = 0.0034 and *P* = 0.0012, respectively; Fig. 3l) could reflect the reduced swimming activity of fish at low temperature. By contrast, 21-d chronic exposure at 34 °C showed a reduction in the time and distance spent in the social area^56^. This result suggests that the stress condition induced by 4-d heat treatment at 34 °C does not alter the interest in conspecifics; however, prolonged exposure to the same temperature for three weeks causes an alteration of the social interaction.

### Mirror biting test

In the MBT (Fig. 4a), no significant differences were observed in the time spent (Fig. 4k) and distance travelled (Fig. 4l) in the mirror approach zone and mirror bites (Fig. 4m) among the three temperatures. The significant reduction in the entries in the mirror zone (*P* < 0.0001 and *P* = 0.0001, Fig. 4j) and increase in the mirror biting latency (*P* = 0.0384, Fig. 4n) observed at 18 °C versus 34 °C could reflect the reduced swimming activity of fish at low temperature.

Again, we observed a difference in behavioural results between acute and chronic exposure at 34 °C: 21-d exposure caused a reduction in the number of bites and, thus, a reduced exhibition of intraspecific aggressive patterns^56^, while the 4-d exposure did not alter the number of bites.

This finding highlights that the effect of high temperatures on behaviour depends on the duration of the exposure and that some alterations observed after chronic exposure are not yet detectable or significant after acute exposure.

### Y-Maze

The YMT was used to evaluate the tendency of the animals to explore the new environment and assess whether the exploratory behaviour is modulated by past experiences (Fig. 5a). The exploration of the new environment constituted by the N arm was evaluated in Te by analysing the time spent in the N arm (Fig. 5j) and the number of entries in the N arm (Fig. 5k) and in S3 of the N arm (Fig. 5l) in the four trials (T1–T4). The results of two-way repeated measures ANOVA with Bonferroni post hoc analysis revealed a significant reduction in the time spent in the N arm from T1 to T3 and from T1 to T4 (*P* = 0.0141 and *P* = 0.0152, respectively; Fig. 5j), in the number of entries in the N arm from T1 to T4 (*P* = 0.0005, Fig. 5k) and in the number of entries in S3 from T1 to T3, from T1 to T4 and from T2 to T4 (*P* = 0.0008, *P* < 0.0001 and *P* = 0.0102, respectively; Fig. 5l) in zebrafish at 26 °C. No differences were observed at 18 °C and 34 °C.

The data at 26 °C confirm previous results^14^ demonstrating that zebrafish at the control temperature (26 °C) modulate the explorative behaviour by progressively reducing their interest for the N arm based on previous experience. These results also confirm that zebrafish at 26 °C maintain their ability in spatial orientation, distinguishing the unexplored from the explored arms and keeping memories of past experiences, as demonstrated by the reduced interest for the N arm when already explored.

By contrast, the experimental subjects at 18 °C and 34 °C did not modulate or modify exploratory behaviour from T1 to T4, suggesting that their cognitive abilities are altered by the acute thermal treatment.

The cause of this alteration can be different in subjects at 18 °C and 34 °C. No difference in locomotor parameters that could affect exploratory behaviour was observed between 34 and 26 °C, suggesting that the altered exploration may be due to cognitive impairment.

Conversely, the reduction in locomotor parameters (such as average speed and total distance travelled) and the increase in freezing events observed in subjects at 18 °C compared to 26 °C can influence the exploratory behaviour of zebrafish and can explain the reduced number of entries in the new arm observed at 18 °C. However, it cannot be excluded that the differences in exploration observed at 18 °C versus to 26 °C also reflect cognitive impairment. In fact, no significant differences in the locomotor parameters were observed at 18 °C among trials (Table S7), indicating that the experimental subject had a similar exploration capacity in all trials. Consequently, the reduced locomotor parameters would not prevent the zebrafish from spending more time in the N arm if interested in the novel environment.

Interestingly, similar results were obtained in adult zebrafish subjected to the same behavioural paradigm^14^ after 21 d of acclimatisation to the same temperatures.

Therefore, the exposure of the zebrafish at 18 °C or 34 °C for 4 d causes an alteration of their exploratory behaviour that persists after 21 d, suggesting that acute thermal treatment induces cognitive impairments that are not recovered during chronic exposure.

### Proteomic analysis

Proteomic analysis from the brain of adult zebrafish kept at 18 °C or 34 °C for 21 d clearly showed that the two experimental temperatures induced differential expression of proteins involved in important cellular events—e.g., cytoskeletal organisation, mitochondria regulation and energy metabolism—and reduced the expression of proteins involved in cognitive processes, synapses and neurotransmitter release, compared with control fish at 26 °C^14^.

In the present study, the same quantitative shotgun approach was applied to the whole brain of zebrafish kept for 4 d at 18 °C, 34 °C and 26 °C. This approach enabled the detection of 1592 total brain proteins at 18 °C and 1470 at 34 °C against 1595 total proteins expressed at 26 °C.

**Figure 6:**
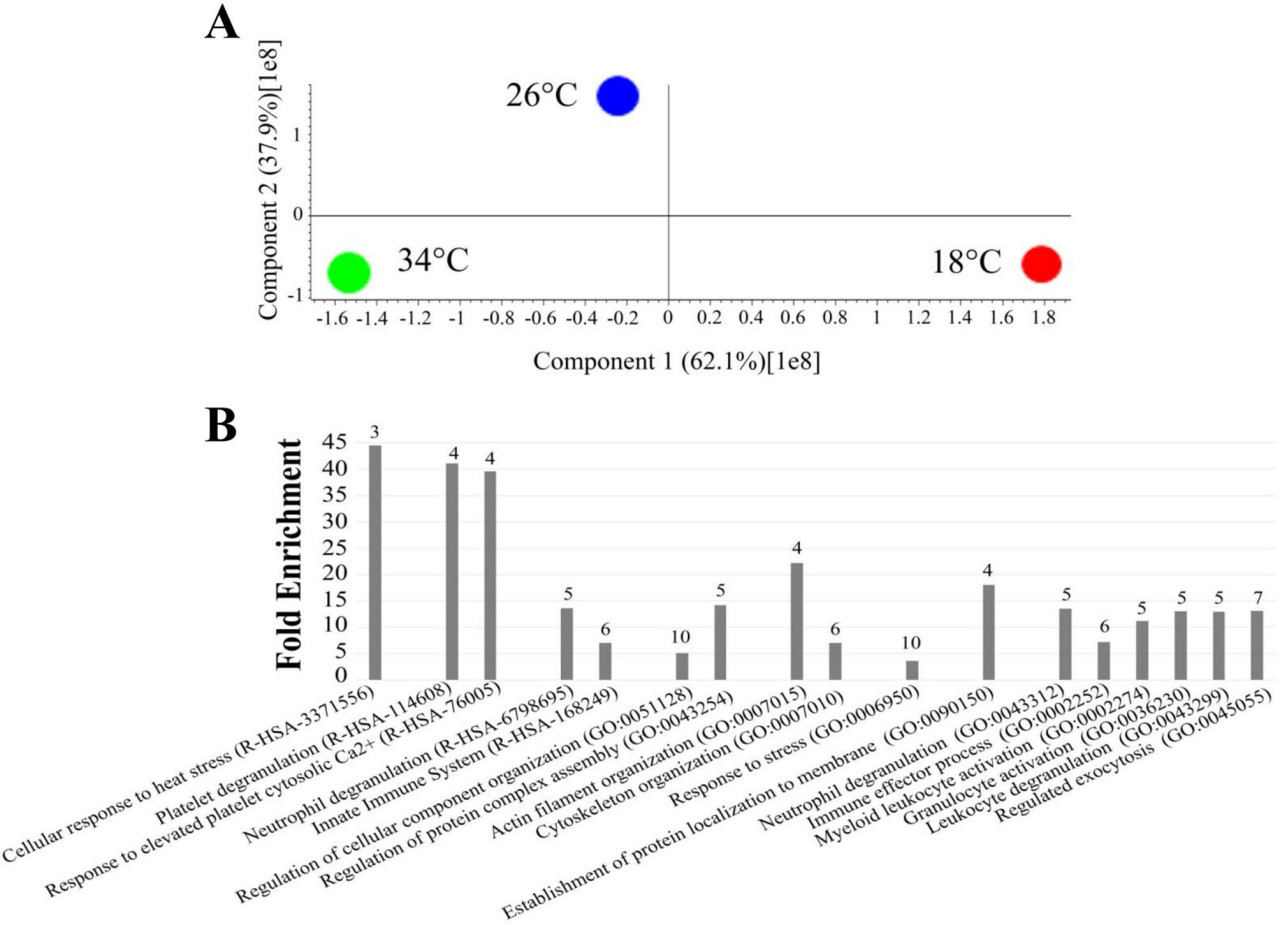
PCA and Panther analysis of the proteome of zebrafish kept at 18 °C, 34 °C and 26 °C. (**A**) PCA analysis of the proteomes of zebrafish kept for 4 days at 18 °C, 26 °C and 34 °C. (**B**) Panther analysis of the proteins differentially expressed among the three data set: 18 °C, 26 °C and 34 °C. The numbers on top of each column indicates the number of genes present in each category. Functional grouping was based on *P* ≤ 0.05 and at least three counts.

PCA analysis carried out to identify major differences among the three datasets highlights differences among the two proteomes under stress conditions that are in contrast to the comparison at 26 °C but are also different from each other (Fig. 6a).

A comparison of the three proteomes by ANOVA (FDR 0.01) shows proteins exclusively expressed at each temperature, as well as 1334 proteins present at all temperatures, among which 18 are differentially expressed under the thermal conditions tested. Bioinformatic analysis of these proteins carried out by Panther (Fig. 6b) highlights that at 34 °C, 18 °C and 26 °C the proteome is different suggesting that thermal regime has a significant impact on cellular and cytoskeleton organisation, stress and the immune system response.

**Figure 7:**
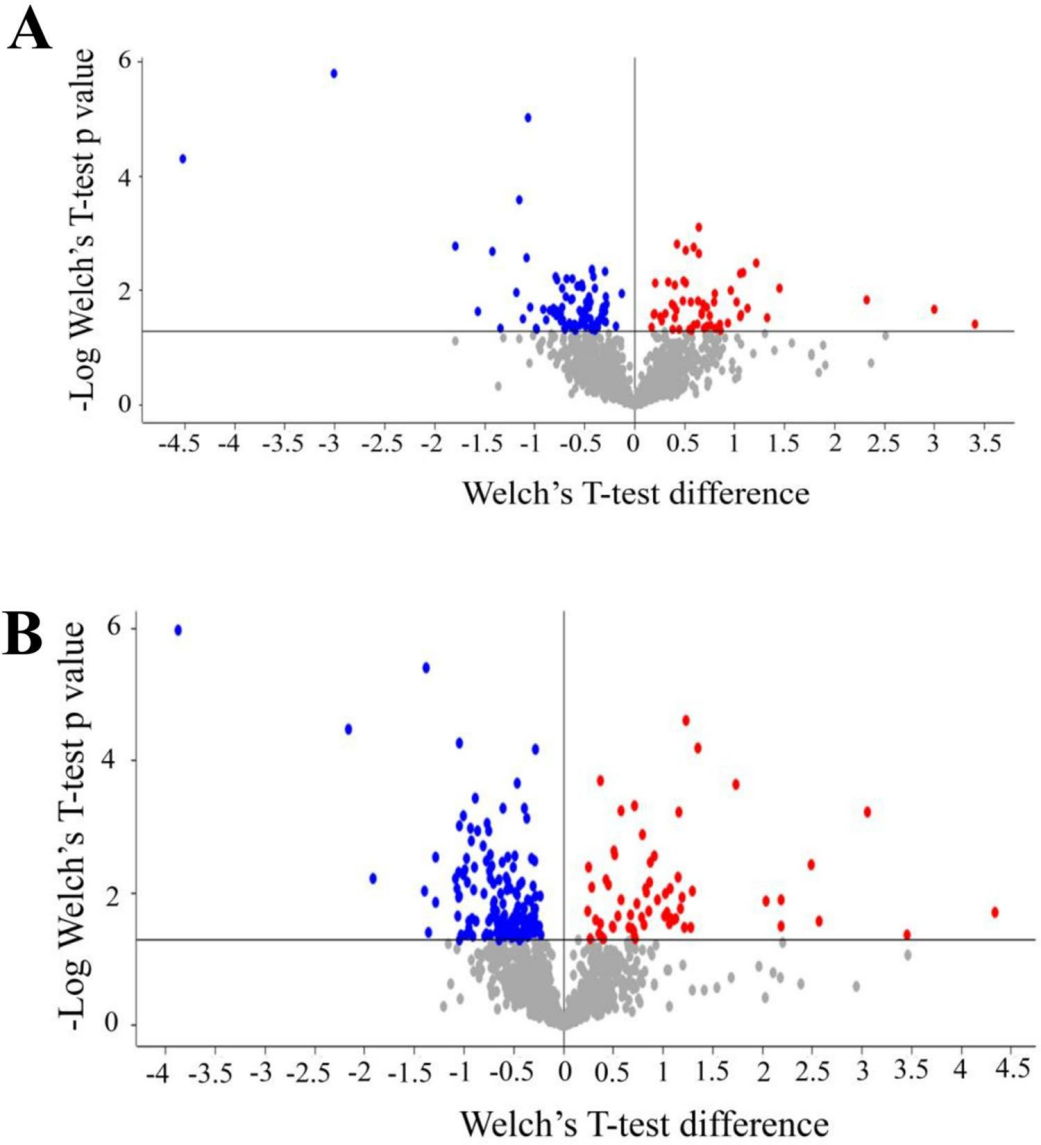
Volcano plots comparing the proteome of zebrafish at 18 °C and 34 °C with 26 °C, kept as the control condition. Volcano plots of the proteins differentially expressed in the comparison 18 °C versus 26 °C (**A**) and 34 °C versus 26 °C (**B**), respectively. Proteins were considered differentially expressed if showed significant t-test difference (Welch's test *P* ≤ 0.05). Proteins more expressed at stress temperatures (18 °C or 34 °C) compared to control are shown in blue while proteins less expressed in stress conditions are shown in red.

**Figure 8:**
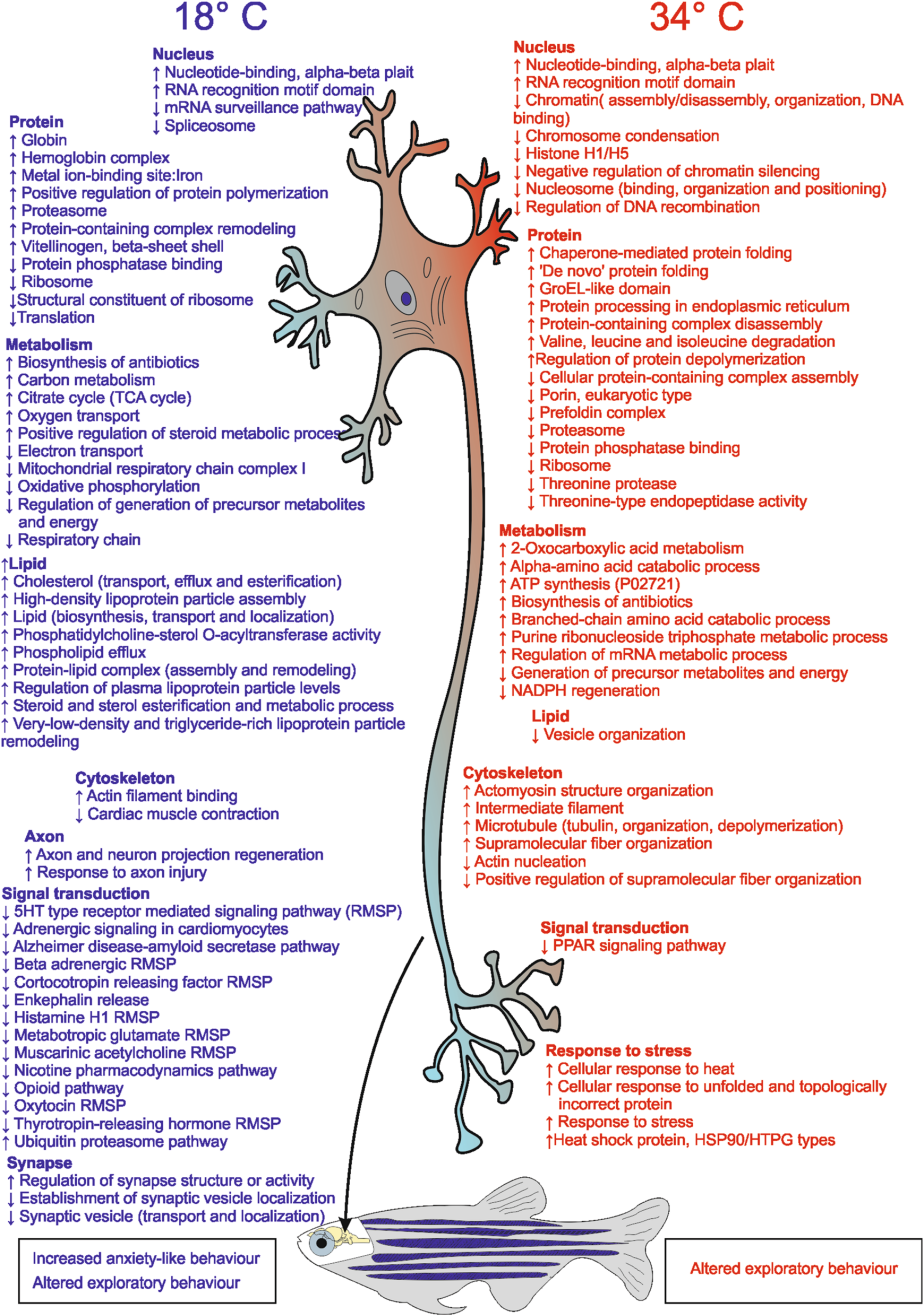
Schematic summary of Gene Ontology classification of brain proteins expressed after thermal treatment fully reported in Tables 1, S2–S16. Down regulation ↓, Up regulation ↑ and general variation ↑↓ of proteins are reported at 18 °C on the left and at 34 °C on the right.

As in the previous study, specific analyses were carried out by comparing 18 °C versus 26 °C and 34 °C versus 26 °C. Tables S8–S11 present the list of proteins that are differentially expressed (either increased or decreased) or exclusively expressed in one condition, while the corresponding volcano plots are shown in Fig. 7. These proteins were further analysed using different software programs (David, Panther, Cluego) to identify possible enrichment in GO terms and pathways. The results were compared with similar analyses carried out on zebrafish subjected to 21-d chronic thermal treatment at identical temperatures^14^, as reported in Tables S12 (Panther analysis), S13, S14 (David analysis) and S15, S16 (Cluego analysis) and in Table 1 and Fig. 8, which summarise the main bioinformatic results.

**Table 1:** Summary of the main findings from bioinformatic analysis by Panther, David and Cluego software programs.

18 °C		34 °C	
Acute	Chronic	Acute	Chronic
**Decreased**			
Oxidative Phosphorylation	Oxidative PhosphorylationATPsynthesis coupled proton transport		Oxidative Phosphorylation
Synaptic vesicle transport	Synaptic vesicle	Synaptic vesicle	Synaptic vesicle
Ribosome, Splicing, Translation	Ribosome, Splicing, Translation	Ribosome	Ribosome
Opioid, adrenergic, metabotropic-glutamate e 5-HT signalling	Opioid, adrenergic, metabotropic-glutamate e 5-HT signalling	PPAR signalling pathway	PPAR signalling pathway
Adrenergic signalling	Adrenergic signalling	Proteasome	Proteasome
	EGF/FGF signalling		EGF/FGF signalling
	Cys/Met metabolism		CH_3_ groups metabolism
	Proteasome		Ca^2+^
	Neuronal ion channel clustering		Fructose and Mannose metabolism
			Integrin signalling
		Porin, Anion transport	
		Chromatin organization	
**Increased**			
TCA Cycle	TCA Cycle	Stress response/heat shock/chaperonins	Stress response/heat shock/chaperonins
Ubiquitin/proteasome	Ubiquitin/proteasome	Intermediate filament	Intermediate filament
Oxygen transport		Actomiosin structure organization	Actomiosin structure organization
Lipid Transport/cholesterol efflux		Microtubule cytoskeleton organization	
Axon regeneration		Protein processing in RE/ folding	
	Integrin signalling	ATP synthesis	
	CCKR signalling	Branched-chain amino acid catabolism	
	Glycolysis/carbohydrates metabolism		Lipid transport
	Pentose Phosphate pathway		Wnt signalling
			Inflammation chemokine/cytokine
			Cadherin signalling

The analysis was carried out on the proteins differentially expressed (either up- or downregulated) or exclusively expressed under stress conditions with 26 °C as the control. Chronic refers to zebrafish kept at 18 °C or 34 °C for 21 d (mass spectrometric data published previously^14^), while acute refers to zebrafish kept at the same temperatures for 4 d. Protein classification is based on GOBP, GOMF, KEGG and Pathway with *P* ≤ 0.05 and a minimum of 3 counts.

As shown in Table S12, the modulation of Panther pathways by 4-d acute thermal treatment is different at low and high temperatures. Several pathways are downregulated in the comparison at 18 °C versus 26 °C, while no statistically significant enrichment of specific Panther pathways was detected between 34 and 26 °C. This finding suggests that the acute exposure impacts more at low than at high temperatures, consistent with the NTT, LDT, SPT and MBT behavioural results revealing significant differences in the comparison 18 °C versus 26 °C but not in 34 °C versus 26 °C. Although any correlation between the variations detected by Panther analysis and the observed behavioural alterations must be considered merely speculative, we can hypothesise that the downregulation of defined pathways at 18 °C is responsible for the increase in anxiety-like status and consequent alterations in behaviour.

The reduction in total distance travelled by fish at 18 °C is consistent with the downregulation of histamine H1 and serotonin receptor-mediated signalling pathways because it has been shown that locomotor activity and exploratory behaviour^82^ are impaired in mice lacking histamine H1 receptors and in zebrafish larvae exposed to an inverse agonist of the H1 receptor^83,84^. Moreover, serotonin depletion was found to reduce explorative locomotion in rats^85^. The downregulation of the serotonin pathway could also contribute to the increased anxiety-like behaviour shown by fish at 18 °C in the NTT, as several reports have shown that acute pharmacological increases in the 5-HT levels decrease anxiety-like behaviour in the NNT^86–89^.

The anxiolytic action of oxytocin is well documented in both rodents and humans^90–92^; thus, the downregulation of the oxytocin receptor-mediated signalling pathway could contribute to the anxiety-like behaviour observed in zebrafish at 18 °C. Interestingly, the downregulation of oxytocin and serotonin pathways could be related to each other because oxytocin in mice regulates serotonin release and exerts anxiolytic effects via direct activation of oxytocin receptors in serotonergic neurons of the raphe nuclei^93^.

In NTT-based literature studies, lower levels of delta opioids were found to be expressed in shy than in bold adult zebrafish^94^. Consistently, the present results showed that the reduction in the enkephalin release and opioid pathways at 18 °C is associated with minor boldness. The downregulation of the opioid pathway could also impair C-start escape responses in zebrafish at 18 °C because opioids potentiate electrical transmission on Mauthner cells^95^.

The anxiety-like levels recorded at 18 °C could also be related to the downregulation of the muscarinic acetylcholine receptor (mAChR) and nicotine pharmacodynamics signalling pathways because experiments conducted using mAChR agonists and antagonists demonstrated that endogenous Ach reduces anxiety through actions on nicotinic and muscarinic receptors whereas mAChR antagonists induce anxiogenic responses in rats^96,97^.

Finally, the downregulation of the **t**hyrotropin-releasing hormone receptor (TRH-R) signalling pathway could contribute to the anxiety detected in zebrafish at 18 °C because TRH-R1-deficient mice display increased anxiety-like behaviour^98^.

The comparison between the 4-d acute and 21-d chronic thermal treatments showed that the Panther pathways are modulated by low temperature similarly in chronic and acute conditions. In both conditions, opioid, metabotropic glutamate receptor, beta-adrenergic receptor and 5HT receptor signalling are decreased at 18 °C whereas the cytoskeleton seems to be mainly affected in chronic stress and the ubiquitin–proteasome pathway is mainly increased in the acute response (Table S12).

Several differences were noted between the acute and chronic treatments at 34 °C with both increases or decreases in numerous pathways with chronic treatment and only increases in others (ATP synthesis) with acute treatment.

The results were generally confirmed by DAVID analysis (Table S13,S14). At low temperature (18 °C), the major difference between the chronic and acute treatments is the significant decrease in oxidative phosphorylation and electron transport under acute conditions but increase in TCA cycle activities under both conditions suggests that, at low temperature, this cycle not only plays a role in redox processes but is also very important to produce metabolites controlling cell fate and function^99^. TCA metabolites are involved in the regulation of chromatin modifications and DNA methylation and cell protein expression, consistent with the enrichment in the RNA translation and ribosome categories at 18 °C (Table S13)^99^. Low temperature also disrupts folding and cysteine and methionine metabolism with chronic treatment; however, with acute treatment, the growth at 18 °C bursts oxygen and lipid transport probably to fuel the TCA cycle and alter processes involving methyl groups (Table S13).

At high temperature (34 °C), anion and porin transport are specifically decreased under acute compared with chronic conditions; however, PPAR signalling decreased under both acute (DAVID, Table S14) and chronic exposure (Cluego, Table S16). PPAR signalling plays a modulatory role in the expression of genes involved in lipid metabolism, adipogenesis, maintenance of metabolic homeostasis, and inflammation^100^. It is now known that adipose tissue participates in mediating the susceptibility to stress and emotion-related behaviours by responding to PPAR signalling and secreting adipokines that operate as hormones in the brain crossing the blood–brain barrier^101^. Their decrease is strictly related to the susceptibility to stress and depression/anxiety-related behaviours.

The impact of acute heat stress on anion transport and porins is mainly due to the decreased expression of voltage-dependent anion channel VDAC proteins of the mitochondrial outer membrane that regulate the influx of respiratory substrates ADP and Pi into the mitochondrion and ATP release into the cytosol under both physiological and pathological conditions. In many eukaryotic cells, VDACs are involved in maintaining cell homeostasis under different stress responses, such as oxidative, salt and temperature stress^102^. In mice, decreased VDAC expression is associated with decreased ATP synthesis and deficits in learning and synaptic plasticity^103^. Interestingly, in the fish brain at 34 °C, there is an increase (Table S14) in microtubule-based processes, tubulin and intermediate filament, which may potentiate the negative effect of heat stress on VDAC, as reported previously^104^ where high free tubulin in cancer cells contributes to close VDAC. These phenomena limit the exchange of respiratory substrates and ATP and decrease the mitochondrial membrane potential, which modulate mitochondrial metabolism, ROS formation, and the intracellular flow of energy.

The chronic and acute datasets were then analysed by Cluego, which allows protein clusterisation based on GOBP, GOMF and KEGG with *P* ≤ 0.05 and a minimum of 3 counts. The results mirrored what was already observed by Panther and David pointing out some interesting aspects in the comparison (Table S15, S16).

Low temperature (18 °C) under acute conditions significantly increased the expression of proteins involved in the regulation of synapse structure, response to axon injury and axon regeneration. The response to oestrogen stimulation and lipid transport is also increased in accordance with Wang et al.^105^ who described the upregulation of genes related to lipid transport in transgenic zebrafish acclimated at low temperature. Under both chronic and acute exposure, cold temperature decreases oxidative phosphorylation and transmission by synaptic vesicles.

At high temperature (34 °C), acute stress induces proteins related to microtubule cytoskeleton organisation and chaperone-mediated protein folding, decreasing nucleosome and vesicle organisation.

The major bioinformatic results summarised in Table 1 suggest that thermal treatment, either high or low temperature, regardless of chronic or acute, has a major impact on energy metabolism, translation, protein synthesis, folding and degradation, cytoskeleton organisation and synaptic vesiculation. The reduction of oxidative phosphorylation (OXPHOS) with thermal treatment and increased glycolysis following low-temperature adaptation agree with the results recently reported in C2C12 myoblasts that prefer glycolysis as a rapid compensatory mechanism to meet the energy requirements for adaptive thermogenic response and reduce aerobic respiration following exposure to cold or heat stress. In C2C12 cells, this effect is linked to epigenetic changes (histone H4 acetylation and global DNA methylation) observed under both heat and cold stress^106^. Concurrent with a metabolic shift, in chronically exposed zebrafish, we observed a decrease in CH3 groups at 34 °C and in Cys/Met metabolism at 18 °C, suggesting that methylation processes could be affected by thermal stress possibly involving alteration in DNA methylation, nucleosome assembly and chromatin organisation (Tables 1, S13 and S16). These observations could also underline that, in zebrafish, crosstalk exists between alteration in energy metabolism and epigenetics during the stress response, leading to behaviour alteration after prolonged exposure^106^.

The impact of thermal treatment on protein synthesis and folding, as reported in Table 1, agrees well with extensive literature reporting that the stress response typically affects protein synthesis and increases chaperone-mediated protein folding, mainly at high temperature^107^. Overall, this response leads to stress of the endoplasmic reticulum (ER), a central organelle that regulates stress responses in both plant and animal cells^108^. The stresses affecting protein folding lead to ER stress, which is communicated to the nucleus via the unfolded protein response (UPR), a cellular homeostatic response to ER stress that is reported to be associated with translation attenuation and psychiatric disorders in humans^109^.

Remarkable is the decrease in EGF/FGF signalling following long-term thermal exposure considering that FGF decrease has been associated with multiple neurological disorders^110^. However, in our model, the impact on this pathway can be observed only under chronic conditions (Table 1) and is not counted in the events that the cells launch immediately upon thermal variation, in contrast to the metabolic shift and ER involvement that are also observed after several days of acclimatization.

The effects of thermal stress on the cytoskeleton were already underlined by the experiments conducted on zebrafish following 21-d exposure to 18° or 34 °C^14^, and the present data confirmed the observation under acute conditions at both 18° and 34 °C. Previous data in the literature have reported that neuronal cells change their growth properties in response to external physical stimuli, such as variations in external temperature, stiffness of the growth substrate, or topographical guidance cues^111^. The structural and mechanical properties of neurons are controlled by the cytoskeleton, a deformable and dynamic biopolymer network comprising actin fibres, intermediate filaments, and microtubules. The cellular cytoskeleton undergoes structural changes and reorganisation in response to external stimuli such as temperature variations that may have a strong impact on neurons. More precisely, Sunnerberg et al.^111^ demonstrated that ambient temperature affects the volume and mechanical properties of cortical neuronal cells at both the bulk (elastic modulus) and local (elasticity maps) levels. In a previous study, the same authors reported that neurons display a significant increase in the average elastic modulus following a decrease in ambient temperature due to the stiffening of the actin components of the cytoskeleton induced by myosin II and molecular motors. Additionally, during phases of active neurite extension, the soma of cortical neurons stiffens reversibly due to changes in microtubule aggregation that differ with the temperature^26^. Literature findings have demonstrated variations in the soma volume and elasticity of cortical neurons in response to changes in cytoskeleton dynamics (microtubules and actin) at different temperatures. In fish brains, motor proteins increase following chronic exposure at 34 °C; in the acute response, the increase prevails in microtubule-based processes and intermediate filaments.

We may tentatively speculate that the behavioural changes observed after acute and chronic treatment at both low and high temperature—i.e., impairment of cognitive abilities associated with spatial orientation, alteration of interest in the new environment or reduced ability to recognise geometric shape—may be due to changes in the neuronal cytoskeleton and physico-chemical properties of neurons at different temperatures. Consistent with this hypothesis, recent studies also suggest that microtubule dynamics in the adult brain plays a pivotal role in the essential processes of learning and memory and may be compromised in neurodegenerative diseases^112^.

Changes in cytoskeleton dynamics also have a significant impact on axon migration and axonal transport, which are decreased in thermal stressed brains under acute and chronic conditions, altering the endo- and exocytosis of synaptic vesicles.

Notably, a significant increase was observed in proteins involved in lipid transport and cholesterol efflux after 4 d at 18 °C and in vitellogenin involved in fat mobilisation following acclimatization at 34 °C for 21 d.

Both processes can be activated in response to oestrogen/oestradiol stimulation under physiological conditions. It is well known that temperature may be sensed by thermotropic changes in macromolecular structures not only through conformational changes in proteins or RNA but also through the microdomain organisation of membrane lipids. Poikilothermic organisms can compensate cellular disturbances induced by temperature fluctuation through physiological and biochemical mechanisms of homeoviscous adaptation by adjusting their membrane lipid composition to maintain fluidity^113^. Lowering the temperature causes an increase in the membrane lipid order, which could cause cholesterol efflux, as observed in zebrafish kept at 18 °C (Table 1). Under heat stress, zebrafish must counteract major membrane fluidity using a long process, over many days, causing the binding to specific Hsps associated transiently with the membrane^113^. A concomitant increase in the lipid transport protein vitellogenin was found in zebrafish at 34 °C (Table 1). This protein is involved in membrane homeostasis in *C. elegans* and other eukaryotes^114^. Membrane fluidity is very important for neuron plasticity, as reported by Chauve et al. who recently described a thermostat-based mechanism that centrally coordinates membrane fluidity in response to warm temperatures across tissues in multicellular animals^115^. Membrane homeostasis plays an important role in synaptogenesis and neuronal differentiation, thus facilitating the normal development of cognitive abilities, while alterations in membrane lipid composition can lead to membrane instability and synaptic loss found in many neurological disorders such as AD^116^.

## Conclusions

Behavioural tests conducted on adult zebrafish subjected to 4-d acute thermal treatment at 18 °C, 26 °C and 34 °C demonstrate increased anxiety-like status at 18 °C compared with controls (26 °C) and a higher temperature (34 °C) and impaired cognitive abilities at both 18 °C and 34 °C compared with 26 °C. Brain proteomic analysis showed altered protein expression at 18 °C and 34 °C compared with the controls (26 °C) and these neurochemical alterations could cause the recorded behavioural coping response observed.

Regarding anxiety levels, behavioural patterns associated with the reduction of the animal's boldness, such as a higher number of freezing events, greater tendency to rest at the bottom of the tank in the NTT^38,60,62,74,81,117^, and preference for dark and protected areas in the LDT^60,61^, were observed at 18 °C but not at 34 °C, compared with 26 °C. Thus, 4-d thermal treatment at 18 °C impacts zebrafish behaviour more than that at 34 °C. Similarly, Panther analysis showed downregulation of proteins involved in anxiety-related signalling pathways at 18 °C but not at 34 °C, compared with 26 °C, suggesting that neurochemical alterations induced by low temperature can contribute to the increase in anxiety-like behaviour. Therefore, the results reported here and in our previous works^56^ show that low and high temperatures have opposite effects on zebrafish anxiety. The 4-d acute treatment at 18 °C increases anxiety-like behaviour, while the 21-d chronic exposure to 34 °C reduces anxiety-like behaviour and increases pro-active behaviour and boldness.

Concerning cognitive performance, the results of the YMT demonstrate that both 4-d acute and 21-d chronic thermal treatments at 18 °C and 34 °C alter the exploratory behaviour, which does not seem to be modulated by past experiences, suggesting an impairment of cognitive abilities. Our proteomic analysis suggests that this impairment may be explained by the alteration of energy metabolism, cytoskeleton organisation, synaptic vesiculation and membrane lipid composition, which occur after short-term exposure but continues even after long periods of exposure.

This work shows that although zebrafish is a model species characterised by wide thermal tolerance, both acute and chronic thermal variations within its vital range alter brain protein expression, cognitive abilities and other behavioural features. Protein expression is altered differently under acute and chronic treatments at both high and low temperatures. This finding suggests that the acclimatization process involves the expression of different proteins and biochemical response begins after acute stress conditions. Both acute and chronic thermal treatments induce alterations in zebrafish behaviour. In particular, exploratory behaviour is already affected after 4 d and the observed alterations persist after 21 d of heat treatment, suggesting that three weeks of acclimatization are not sufficient to restore fish cognitive abilities. Therefore, even short-term thermal variations can alter the ability of zebrafish to face challenges and may compromise its survival in the natural environment.

## Supplementary Information

Supplementary Tables S1–S7.

Supplementary Tables S8–S16.

## Data Availability

The behavioural data have been deposited on *figshare* repository (10.6084/m9.figshare.12382496). The mass spectrometry proteomics raw data have been deposited to the ProteomeXchangeConsortium via the PRIDE^72^ partner repository with the dataset identifier PXD016847.

## References

[1] Brett JR (1971). Energetic responses of salmon to temperature. a study of some thermal relations in the physiology and freshwater ecology of sockeye salmon (*Oncorhynchus nerkd*). Integr. Comp. Biol..

[2] Beitinger TL, Bennett WA, McCauley RW (2000). Temperature tolerances of North American freshwater fishes exposed to dynamic changes in temperature. Environ. Biol. Fish.

[3] Daufresne M, Lengfellner K, Sommer U (2009). Global warming benefits the small in aquatic ecosystems. Proc. Natl. Acad. Sci. USA.

[4] Somero GN (2004). Adaptation of enzymes to temperature: Searching for basic "strategies". Comp. Biochem. Physiol. B Biochem. Mol. Biol..

[5] Houlihan, D. F., Mathers, E. M. & Foster, A. In: *Fish Ecophysiology* (eds. J. Cliff Rankin & Frank B. Jensen) 45–71 (Springer, Netherlands, 1993).

[6] Britz PJ, Hecht T, Mangold S (1997). Effect of temperature on growth, feed consumption and nutritional indices of *Haliotis midae* fed a formulated diet. Aquaculture.

[7] Azevedo PA, Cho CY, Leeson S, Bureau DP (1998). Effects of feeding level and water temperature on growth, nutrient and energy utilization and waste outputs of rainbow trout (*Oncorhynchus mykiss*). Aquat. Living Resour..

[8] Stoner AW (2004). Effects of environmental variables on fish feeding ecology: Implications for the performance of baited fishing gear and stock assessment. J. Fish Biol..

[9] Claireaux G, Couturier C, Groison AL (2006). Effect of temperature on maximum swimming speed and cost of transport in juvenile European sea bass (*Dicentrarchus labrax*). J. Exp. Biol..

[10] Sfakianakis D (2006). Environmental determinants of haemal lordosis in european sea bass, *Dicentrarchus labrax* (Linnaeus, 1758). Aquaculture.

[11] Malavasi S (2013). Effects of temperature on the antipredator behaviour and on the cholinergic expression in the European sea bass (*Dicentrarchus labrax* L.) juveniles. Ethology.

[12] Manciocco A (2015). The acclimation of european sea bass (*Dicentrarchus labrax*) to temperature: Behavioural and neurochemical responses. Ethology.

[13] Toni M, Angiulli E, Malavasi S, Alleva E, Cioni C (2017). Variation in environmental parameters in research and aquaculture: Effects on behaviour, physiology and cell biology of teleost fish. J. Aquac. Mar. Biol..

[14] Toni M (2019). Environmental temperature variation affects brain protein expression and cognitive abilities in adult zebrafish (*Danio rerio*): A proteomic and behavioural study. J. Proteomics.

[15] Prokkola JM (2018). Cold temperature represses daily rhythms in the liver transcriptome of a stenothermal teleost under decreasing day length. J. Exp. Biol..

[16] Lyu L (2018). Deep transcriptomic analysis of black rockfish (*Sebastes schlegelii*) provides new insights on responses to acute temperature stress. Sci. Rep..

[17] Brijs J (2018). In vivo aerobic metabolism of the rainbow trout gut and the effects of an acute temperature increase and stress event. J. Exp. Biol..

[18] Brandão ML (2018). Water temperature affects aggressive interactions in a neotropical cichlid fish. Neotrop. Ichthyol..

[19] Jerônimo R (2017). Thermal stress in *Danio rerio*: a link between temperature, light, thermo-TRP channels, and clock genes. J. Therm. Biol..

[20] Dalvi RS (2017). Metabolic and cellular stress responses of catfish, *Horabagrus brachysoma* (Gunther) acclimated to increasing temperatures. J. Therm. Biol..

[21] Chauhan NR (2017). Heat stress-induced neuroinflammation and aberration in monoamine levels in hypothalamus are associated with temperature dysregulation. Neuroscience.

[22] Logan CA, Buckley BA (2015). Transcriptomic responses to environmental temperature in eurythermal and stenothermal fishes. J. Exp. Biol..

[23] Li AJ, Leung PTY, Bao VWW, Lui GCS, Leung KMY (2015). Temperature-dependent physiological and biochemical responses of the marine medaka *Oryzias melastigma* with consideration of both low and high thermal extremes. J Therm. Biol..

[24] Mininni AN (2014). Liver transcriptome analysis in gilthead sea bream upon exposure to low temperature. BMC Genomics.

[25] Mehlis M, Bakker TCM (2014). The influence of ambient water temperature on sperm performance and fertilization success in three-spined sticklebacks (*Gasterosteus aculeatus*). Evol. Ecol..

[26] Spedden E, Kaplan DL, Staii C (2013). Temperature response of the neuronal cytoskeleton mapped via atomic force and fluorescence microscopy. Phys. Biol..

[27] Madeira D, Narciso L, Cabral HN, Vinagre C, Diniz MS (2013). Influence of temperature in thermal and oxidative stress responses in estuarine fish. Comp. Biochem. Phys. A.

[28] 28Currie, S. & Schulte, P. M. Neuronal regeneration. In: *The physiology of fishes* (eds. Evans D.H., Claiborne J.B., & Currie S.) 257–287 (CRC Press, 2014).

[29] Dhabhar FS (2000). Acute stress enhances while chronic stress suppresses skin immunity. The role of stress hormones and leukocyte trafficking. Ann. NY Acad. Sci..

[30] Wang D (2020). Behavioral and physiological effects of acute and chronic kava exposure in adult zebrafish. Neurotoxicol. Teratol..

[31] Rossi A, Bacchetta C, Cazenave J (2017). Effect of thermal stress on metabolic and oxidative stress biomarkers of *Hoplosternum littorale* (Teleostei, Callichthyidae). Ecol. Indic..

[32] Lermen CL (2004). Effect of different temperature regimes on metabolic and blood parameters of silver catfish *Rhamdia quelen*. Aquaculture.

[33] Barreto RE, Volpato GL (2004). Caution for using ventilatory frequency as an indicator of stress in fish. Behav. Process..

[34] McCormick SD (1998). Repeated acute stress reduces growth rate of Atlantic salmon parr and alters plasma levels of growth hormone, insulin-like growth factor I and cortisol. Aquaculture.

[35] Santos GA, Schrama JW, Mamauag REP, Rombout JHWM, Verreth JAJ (2010). Chronic stress impairs performance, energy metabolism and welfare indicators in European seabass (*Dicentrarchus labrax*): the combined effects of fish crowding and water quality deterioration. Aquaculture.

[36] Wendelaar Bonga SE (1997). The stress response in fish. Physiol. Rev..

[37] Baggio S, Mussulini BH, de Oliveira DL, Gerlai R, Rico EP (2018). Embryonic alcohol exposure leading to social avoidance and altered anxiety responses in adult zebrafish. Behav. Brain Res..

[38] Bencan Z, Sledge D, Levin ED (2009). Buspirone, chlordiazepoxide and diazepam effects in a zebrafish model of anxiety. Pharmacol. Biochem. Behav..

[39] Blaser RE, Penalosa YM (2011). Stimuli affecting zebrafish (*Danio rerio*) behavior in the light/dark preference test. Physiol. Behav..

[40] Cachat J (2010). Measuring behavioral and endocrine responses to novelty stress in adult zebrafish. Nat. Protoc..

[41] Cognato G, P. (2012). Y-Maze memory task in zebrafish (*Danio rerio*): The role of glutamatergic and cholinergic systems on the acquisition and consolidation periods. Neurobiol. Learn. Mem..

[42] Barbosa HP, Lima-Maximino MG, Maximino C (2019). Acute fluoxetine differently affects aggressive display in zebrafish phenotypes. Aggress. Behav..

[43] Zimmermann FF, Gaspary KV, Siebel AM, Bonan CD (2016). Oxytocin reversed MK-801-induced social interaction and aggression deficits in zebrafish. Behav. Brain Res..

[44] Chou MY (2008). Effects of hypothermia on gene expression in zebrafish gills: upregulation in differentiation and function of ionocytes as compensatory responses. J. Exp. Biol..

[45] Long Y, Li L, Li Q, He X, Cui Z (2012). Transcriptomic characterization of temperature stress responses in larval zebrafish. PLoS ONE.

[46] Long Y (2013). Transcriptomic characterization of cold acclimation in larval zebrafish. BMC Genomics.

[47] Malek RL, Sajadi H, Abraham J, Grundy MA, Gerhard GS (2004). The effects of temperature reduction on gene expression and oxidative stress in skeletal muscle from adult zebrafish. Comp. Biochem. Physiol. C Toxicol. Pharmacol..

[48] Scott GR, Johnston IA (2012). Temperature during embryonic development has persistent effects on thermal acclimation capacity in zebrafish. Proc. Natl. Acad. Sci. USA.

[49] Vergauwen L, Benoot D, Blust R, Knapen D (2010). Long-term warm or cold acclimation elicits a specific transcriptional response and affects energy metabolism in zebrafish. Comp. Biochem. Physiol. A Mol. Integr. Physiol..

[50] Chakravarty S (2013). Chronic unpredictable stress (CUS)-induced anxiety and related mood disorders in a zebrafish model: Altered brain proteome profile implicates mitochondrial dysfunction. PLoS ONE.

[51] Parichy DM (2015). Advancing biology through a deeper understanding of zebrafish ecology and evolution. eLife.

[52] Spence R, Gerlach G, Lawrence C, Smith C (2008). The behaviour and ecology of the zebrafish, *Danio rerio*. Biol. Rev. Camb. Philos. Soc..

[53] Cortemeglia C, Beitinger TL (2005). Temperature tolerances of wild-type and red transgenic zebra danios. Trans. Am. Fish. Soc..

[54] Schaefer J, Ryan A (2006). Developmental plasticity in the thermal tolerance of zebrafish *Danio rerio*. J. Fish Biol..

[55] López-Olmeda JF, Sánchez-Vázquez FJ (2011). Thermal biology of zebrafish (*Danio rerio*). J. Therm. Biol..

[56] Angiulli E (2020). Increase in environmental temperature affects exploratory behaviour, anxiety and social preference in *Danio rerio*. Sci. Rep..

[57] Toni M (2019). Review: Assessing fish welfare in research and aquaculture, with a focus on European directives. Animal.

[58] Gerlai R, Lahav M, Guo S, Rosenthal A (2000). Drinks like a fish: zebra fish (*Danio rerio*) as a behavior genetic model to study alcohol effects. Pharmacol. Biochem. Behav..

[59] Blaser R, Gerlai R (2006). Behavioral phenotyping in zebrafish: comparison of three behavioral quantification methods. Behav. Res. Methods.

[60] Sackerman J (2010). Zebrafish behavior in novel environments: Effects of acute exposure to anxiolytic compounds and choice of *Danio rerio* line. Int. J. Comp. Psychol..

[61] Maximino C, Marques de Brito T, Dias CA, Gouveia A, Morato S (2010). Scototaxis as anxiety-like behavior in fish. Nat. Prot..

[62] Champagne DL, Hoefnagels CC, de Kloet RE, Richardson MK (2010). Translating rodent behavioral repertoire to zebrafish (*Danio rerio*): Relevance for stress research. Behav. Brain Res..

[63] Oliveira RF (2013). Mind the fish: Zebrafish as a model in cognitive social neuroscience. Front. Neural. Circuit.

[64] Green J (2012). Automated high-throughput neurophenotyping of zebrafish social behavior. J. Neurosci. Methods.

[65] Miller N, Gerlai R (2012). From schooling to shoaling: patterns of collective motion in zebrafish (*Danio rerio*). PLoS ONE.

[66] 66Pham, M. *et al*. Assessing social behavior phenotypes in adult zebrafish: shoaling, social preference, and mirror biting tests. In: *Zebrafish Protocols for Neurobehavioral Research. Neuromethods* (eds. Kalueff A. & Stewart A.) 231–246 (Humana Press, London, 2012).

[67] Norton WH (2011). Modulation of Fgfr1a signaling in zebrafish reveals a genetic basis for the aggression-boldness syndrome. J. Neurosci..

[68] Tedeschi G (2011). Protein pattern of *Xenopus laevis* embryos grown in simulated microgravity. Cell Biol. Int..

[69] Vernocchi V (2014). Sperm ubiquitination in epididymal feline semen. Theriogenology.

[70] Maffioli E (2017). Proteomic dissection of nanotopography-sensitive mechanotransductive signaling hubs that foster neuronal differentiation in PC12 Cells. Front. Cell. Neurosci..

[71] Migliaccio O (2019). Living in future ocean acidification, physiological adaptive responses of the immune system of sea urchins resident at a CO_2_ vent system. Sci. Total Environ..

[72] Tedeschi G (2017). Proteomic profile of maternal-aged blastocoel fluid suggests a novel role for ubiquitin system in blastocyst quality. J. Assist. Reprod. Gen..

[73] Vizcaino JA (2016). 2016 update of the PRIDE database and its related tools. Nucleic Acids Res..

[74] Egan RJ (2009). Understanding behavioral and physiological phenotypes of stress and anxiety in zebrafish. Behav. Brain Res..

[75] Fernandes Y, Gerlai R (2009). Long-term behavioral changes in response to early developmental exposure to ethanol in zebrafish. Alcohol. Clin. Exp. Res..

[76] Buske C, Gerlai R (2011). Early embryonic ethanol exposure impairs shoaling and the dopaminergic and serotoninergic systems in adult zebrafish. Neurotoxicol. Teratol..

[77] Seguin D, Shams S, Gerlai R (2016). Behavioral responses to novelty or to a predator stimulus are not altered in adult zebrafish by early embryonic alcohol exposure. Alcohol. Clin. Exp. Res..

[78] Miller NY, Gerlai R (2011). Shoaling in zebrafish: What we don't know. Rev. Neurosci..

[79] Saverino C, Gerlai R (2008). The social zebrafish: behavioral responses to conspecific, heterospecific, and computer animated fish. Behav. Brain. Res..

[80] Fu C-W (2021). Exposure to silver impairs learning and social behaviors in adult zebrafish. J. Hazard. Mater..

[81] Levin ED, Bencan Z, Cerutti DT (2007). Anxiolytic effects of nicotine in zebrafish. Physiol. Behav..

[82] Inoue I (1996). Impaired locomotor activity and exploratory behavior in mice lacking histamine H1 receptors. Proc. Natl. Acad. Sci. USA.

[83] Renier C (2007). Genomic and functional conservation of sedative-hypnotic targets in the zebrafish. Pharmacogenet. Genom..

[84] Peitsaro N, Sundvik M, Anichtchik OV, Kaslin J, Panula P (2007). Identification of zebrafish histamine H1, H2 and H3 receptors and effects of histaminergic ligands on behavior. Biochem. Pharmacol..

[85] Dringenberg HC, Hargreaves EL, Baker GB, Cooley RK, Vanderwolf CH (1995). p-chlorophenylalanine-induced serotonin depletion: Reduction in exploratory locomotion but no obvious sensory-motor deficits. Behav. Brain Res..

[86] Stewart A (2011). Pharmacological modulation of anxiety-like phenotypes in adult zebrafish behavioral models. Prog. Neuropsychopharmacol. Biol. Psychiatry.

[87] Stewart AM (2013). Perspectives on experimental models of serotonin syndrome in zebrafish. Neurochem. Int..

[88] Herculano AM, Maximino C (2014). Serotonergic modulation of zebrafish behavior: Towards a paradox. Prog. Neuropsychopharmacol. Biol. Psychiatry.

[89] Singer ML, Oreschak K, Rhinehart Z, Robison BD (2016). Anxiolytic effects of fluoxetine and nicotine exposure on exploratory behavior in zebrafish. PeerJ.

[90] Sabihi S, Durosko NE, Dong SM, Leuner B (2014). Oxytocin in the prelimbic medial prefrontal cortex reduces anxiety-like behavior in female and male rats. Psychoneuroendocrinology.

[91] Mantella RC, Vollmer RR, Li X, Amico JA (2003). Female oxytocin-deficient mice display enhanced anxiety-related behavior. Endocrinology.

[92] Heinrichs M, Baumgartner T, Kirschbaum C, Ehlert U (2003). Social support and oxytocin interact to suppress cortisol and subjective responses to psychosocial stress. Biol. Psychiatry.

[93] Yoshida M (2009). Evidence that oxytocin exerts anxiolytic effects via oxytocin receptor expressed in serotonergic neurons in mice. J. Neurosci..

[94] Thornqvist PO, McCarrick S, Ericsson M, Roman E, Winberg S (2019). Bold zebrafish (*Danio rerio*) express higher levels of delta opioid and dopamine D2 receptors in the brain compared to shy fish. Behav. Brain Res..

[95] Cachope R, Pereda AE (2015). Opioids potentiate electrical transmission at mixed synapses on the Mauthner cell. J. Neurophysiol..

[96] Di Liberto V (2017). Anxiolytic effects of muscarinic acetylcholine receptors agonist oxotremorine in chronically stressed rats and related changes in BDNF and FGF2 levels in the hippocampus and prefrontal cortex. Psychopharmacology.

[97] File SE, Gonzalez LE, Andrews N (1998). Endogenous acetylcholine in the dorsal hippocampus reduces anxiety through actions on nicotinic and muscarinic receptors. Behav. Neurosci..

[98] Zeng H (2007). Thyrotropin-releasing hormone receptor 1-deficient mice display increased depression and anxiety-like behavior. Mol. Endocrinol..

[99] Martinez-Reyes I, Chandel NS (2020). Mitochondrial TCA cycle metabolites control physiology and disease. Nat. Comm..

[100] Fanale D, Amodeo V, Caruso S (2017). The interplay between metabolism, PPAR signaling pathway, and cancer. PPAR Res..

[101] Guo M (2017). Role of the adipose PPARgamma-adiponectin axis in susceptibility to stress and depression/anxiety-related behaviors. Mol. Psychiatry.

[102] Sanyal SK (2020). Arabidopsis mitochondrial voltage-dependent anion channels are involved in maintaining reactive oxygen species homeostasis, oxidative and salt stress tolerance in yeast. Front. Plant Sci..

[103] Levy M, Faas GC, Saggau P, Craigen WJ, Sweatt JD (2003). Mitochondrial regulation of synaptic plasticity in the hippocampus. J. Biol. Chem..

[104] Maldonado EN (2017). VDAC-Tubulin, an anti-warburg pro-oxidant switch. Front. Oncol..

[105] Wang Q, Tan X, Jiao S, You F, Zhang PJ (2014). Analyzing cold tolerance mechanism in transgenic zebrafish (*Danio rerio*). PLoS ONE.

[106] Sajjanar B (2019). Cross-talk between energy metabolism and epigenetics during temperature stress response in C2C12 myoblasts. Int. J. Hyperthermia.

[107] Torrent M, Chalancon G, de Groot NS, Wuster A, Madan Babu M (2018). Cells alter their tRNA abundance to selectively regulate protein synthesis during stress conditions. Sci. Signal.

[108] Park CJ, Park JM (2019). Endoplasmic reticulum plays a critical role in integrating signals generated by both biotic and abiotic stress in plants. Front. Plant Sci..

[109] Mao J, Hu Y, Ruan L, Ji Y, Lou Z (2019). Role of endoplasmic reticulum stress in depression (review). Mol. Med. Rep..

[110] Guillemot F, Zimmer C (2011). From cradle to grave: The multiple roles of fibroblast growth factors in neural development. Neuron.

[111] Sunnerberg JP, Moore P, Spedden E, Kaplan DL, Staii C (2019). Variations of elastic modulus and cell volume with temperature for cortical neurons. Langmuir.

[112] Dent EW (2017). Of microtubules and memory: Implications for microtubule dynamics in dendrites and spines. Mol. Biol. Cell.

[113] Balogh G (2013). Key role of lipids in heat stress management. FEBS Lett..

[114] Bodhicharla R, Devkota R, Ruiz M, Pilon M (2018). Membrane fluidity is regulated cell nonautonomously by *Caenorhabditis elegans* PAQR-2 and its mammalian homolog adipoR2. Genetics.

[115] Chauve L (2019). A neuronal thermostat controls membrane fluidity in *Caenorhabditis elegans*. bioRxiv.

[116] Hussain G (2019). Role of cholesterol and sphingolipids in brain development and neurological diseases. Lipids Health Dis..

[117] Grossman L (2010). Characterization of behavioral and endocrine effects of LSD on zebrafish. Behav. Brain Res..

