# Supplementary material for: Acute environmental temperature variation affects brain protein expression, anxiety and explorative behaviour in adult zebrafish
Source: Sci Rep. 2021 Jan 28;11:2521. doi: 10.1038/s41598-021-81804-5 (PMC7843641; doi:10.1038/s41598-021-81804-5)
Supplement: Supplementary file 1 — Supplementary Tables S1–S7. [file 41598_2021_81804_MOESM1_ESM.docx]

**SUPPLEMENTARY INFORMATION for**

**Acute environmental temperature variation affects brain protein expression, anxiety and explorative behaviour in adult zebrafish**

**S. Nonnis^1,2#^, E. Angiulli^3#^, E. Maffioli^1,4*^, F. Frabetti^5^, A. Negri^1,4^, C. Cioni^3^, E. Alleva^6^, V. Romeo^1^, G. Tedeschi^1,2,4^, M. Toni^3*^**

1 Department of Veterinary Medicine, via dell’Università 6, Lodi – University of Milan, Italy

2 CRC "Innovation for well-beeing and environment” (I-WE) University of Milan, Italy

3 Department of Biology and Biotechnology ‘‘Charles Darwin”, Sapienza University, Rome, Italy

4 CIMAINA, Università degli Studi di Milano, Milano, Italy

5 Department of Experimental, Diagnostic and Specialty Medicine, University of Bologna, Italy

6 Center for Behavioural Sciences and Mental Health, IstitutoSuperiore di Sanità, Rome, Italy

**Current file content:**

**Supplementary Table S1**

**Supplementary Table S2**

**Supplementary Table S3**

**Supplementary Table S4**

**Supplementary Table S5**

**Supplementary Table S6**

**Supplementary Table S7**

**Table S1** Zebrafish mass at the end of the thermal treatment.

| Home-tank | Temperature | Mass mean | S.E.M. |
| --- | --- | --- | --- |
| 1 | 18 °C | 0.56 | 0.02 |
| 2 | 34 °C | 0.51 | 0.02 |
| 3 | 34 °C | 0.52 | 0.03 |
| 4 | 26 °C | 0.47 | 0.02 |
| 5 | 18 °C | 0.51 | 0.03 |
| 6 | 26 °C | 0.50 | 0.01 |

**Table S2** Novel tank diving test: P-values for one-way ANOVA followed by Bonferroni's post hoc test.

| NTT | | | |
| --- | --- | --- | --- |
|  | 26°C vs 18°C | 34°C vs 18°C | 34°C vs 26°C |
| Immobile episodes (N) | 0.0014 | 0.0006 | 1.0000 |
| Time immobile (s) | 0.0002 | 0.0002 | 1.0000 |
| Maximum speed (m/s) | 1.0000 | 1.0000 | 1.0000 |
| Average speed (m/s) | <0.0001 | <0.0001 | 1.0000 |
| Total distance travelled (m) | <0.0001 | <0.0001 | 1.0000 |
| CW rotations (%) | 1.0000 | 1.0000 | 1.0000 |
| Absolute turn angle (deg) | <0.0001 | <0.0001 | 0.9306 |
| Meandering | 0.1027 | 0.1121 | 1.0000 |
| Transitions (N) | <0.0001 | <0.0001 | 0.0553 |
| Latency to enter the top area (s) | 0.0120 | 0.0006 | 0.5545 |
| Time in top areas (%) | 0.0001 | <0.0001 | 0.4683 |
| Distance travelled in top area (m) | 0.0001 | <0.0001 | 0.5464 |
| Transitions to top area (N) | <0.0001 | <0.0001 | 0.1486 |

**Table S3** Dark and light test: P-values for one-way ANOVA followed by Bonferroni's post hoc test.

| DLT | | | |
| --- | --- | --- | --- |
|  | 26°C vs 18°C | 34°C vs 18°C | 34°C vs 26°C |
| Time spent in dark area (%) | 0.0079 | <0.0001 | 0.1661 |
| Transitions (N) | 0.0007 | <0.0001 | 1.0000 |

**Table S4** Social preference test: P-values for one-way ANOVA followed by Bonferroni's post hoc test.

| SPT | | | |
| --- | --- | --- | --- |
|  | 26°C vs 18°C | 34°C vs 18°C | 34°C vs 26°C |
| Immobile episodes (N) | 0.2817 | 0.0364 | 1.0000 |
| Time immobile (s) | 0.2356 | 0.0270 | 1.0000 |
| Maximum speed (m/s) | 0.7358 | 1.0000 | 1.0000 |
| Average speed (m/s) | 0.0417 | 0.0007 | 0.4593 |
| Total distance travelled (m) | 0.0389 | 0.0006 | 0.4579 |
| CW rotations (%) | 1.0000 | 1.0000 | 1.0000 |
| Absolute turn angle (deg) | 1.0000 | 1.0000 | 1.0000 |
| Meandering | 1.0000 | 0.4655 | 0.5940 |
| Time spent in social zone (%) | 1.0000 | 0.6352 | 1.0000 |
| Distance travelled in social area (s) | 1.0000 | 0.8877 | 1.0000 |
| Transitions to social area (N) | 0.0034 | 0.0012 | 1.0000 |
| Rotations (N) | 0.5765 | 0.0555 | 0.8004 |

**Table S5** Mirror biting test: P-values for one-way ANOVA followed by Bonferroni's post hoc test.

| MBT | | | |
| --- | --- | --- | --- |
|  | 26°C vs 18°C | 34°C vs 18°C | 34°C vs 26°C |
| Immobile episodes (N) | <0.0001 | <0.0001 | 1.0000 |
| Time immobile (s) | <0.0001 | <0.0001 | 1.0000 |
| Maximum speed (m/s) | 0.7559 | 1.0000 | 1.0000 |
| Average speed (m/s) | 0.0004 | 0.0001 | 1.0000 |
| Total distance travelled (m) | 0.0004 | 0.0001 | 1.0000 |
| CW rotations (%) | 1.0000 | 1.0000 | 0.6038 |
| Absolute turn angle (deg) | 0.0645 | 0.0033 | 0.8080 |
| Meandering | 0.3405 | 0.4271 | 1.0000 |
| Transitions (N) | 0.0001 | <0.0001 | 1.0000 |
| Mirror approach zone entries (N) | <0.0001 | 0.0001 | 1.0000 |
| Mirror approach zone time spent (s) | 1.0000 | 1.0000 | 1.0000 |
| Mirror approach zone distance travelled (m) | 0.4545 | 0.2236 | 1.0000 |
| Mirror bites (N) | 1.0000 | 1.0000 | 1.0000 |
| Mirror biting latency (s) | 1.0000 | 0.0384 | 0.1578 |

**Table S6** Y-Maze test: P-values for two-way ANOVA followed by Bonferroni's post hoc test.

|  | Intra Tr | | |
| --- | --- | --- | --- |
|  | 18°C vs 26°C | 18°C vs 34°C | 26°C vs 34°C |
| Immobile episodes (N) | 0.8646 | 0.0008 | 0.4341 |
| Time immobile (s) | 0.0014 | <0.0001 | 1.0000 |
| Maximum speed (m/s) | 0.9366 | 1.0000 | 0.7919 |
| Average speed (m/s) | <0.0001 | <0.0001 | 1.0000 |
| Total distance travelled (m) | <0.0001 | <0.0001 | 1.0000 |
| CW rotations (%) | 1.0000 | 0.4678 | 1.0000 |
| Absolute turn angle (deg) | 1.0000 | 1.0000 | 1.0000 |
| Meandering | 1.0000 | 1.0000 | 1.0000 |
|  |  |  |  |
|  | Intra Te | | |
|  | 18°C vs 26°C | 18°C vs 34°C | 26°C vs 34°C |
| Immobile episodes (N) | 0.8646 | <0.0001 | 0.0742 |
| Time immobile (s) | <0.0001 | <0.0001 | 0.2802 |
| Maximum speed (m/s) | 0.0504 | 1.0000 | 0.5178 |
| Average speed (m/s) | <0.0001 | <0.0001 | 1.0000 |
| Total distance travelled (m) | <0.0001 | <0.0001 | 1.0000 |
| CW rotations (%) | 0.1369 | 0.0966 | 1.0000 |
| Absolute turn angle (deg) | 0.0014 | 0.1256 | 1.0000 |
| Meandering | 0.1742 | 0.1288 | 1.0000 |
|  |  |  |  |
|  | Intra temperature (Te vs Tr) | | |
|  | 18°C | 26°C | 34°C |
| Immobile episodes (N) | 0.8646 | 0.8646 | 1.0000 |
| Time immobile (s) | 0.0865 | 1.0000 | 1.0000 |
| Maximum speed (m/s) | 1.0000 | 1.0000 | 1.0000 |
| Average speed (m/s) | 1.0000 | 1.0000 | 1.0000 |
| Total distance travelled (m) | 1.0000 | 1.0000 | 1.0000 |
| CW rotations (%) | 1.0000 | 1.0000 | 1.0000 |
| Absolute turn angle (deg) | 0.8014 | 1.0000 | 1.0000 |
| Meandering | 1.0000 | 1.0000 | 1.0000 |

|  | Inter temperature and phase | | | | | |
| --- | --- | --- | --- | --- | --- | --- |
|  | Te 18°C vs Tr 26°C | Te 18°C vs  Tr 34°C | Te 26°C vs  Tr 18°C | Te 26°C vs  Tr 34°C | Te 34°C vs  Tr 18°C | Te 34°C vs  Tr 26°C |
| Immobile episodes (N) | 0.0025 | <0.0001 | 1.0000 | 0.0008 | 0.0742 | 1.0000 |
| Time immobile (s) | <0.0001 | <0.0001 | 0.2061 | 0.0346 | <0.0001 | 1.0000 |
| Maximum speed (m/s) | 0.5131 | 1.0000 | 0.1102 | 0.0885 | 1.0000 | 1.0000 |
| Average speed (m/s) | <0.0001 | <0.0001 | <0.0001 | 1.0000 | <0.0001 | 1.0000 |
| Total distance travelled (m) | <0.0001 | <0.0001 | <0.0001 | 1.0000 | <0.0001 | 1.0000 |
| CW rotations (%) | 1.0000 | 0.0826 | 0.7068 | 1.0000 | 0.5319 | 1.0000 |
| Absolute turn angle (deg) | 0.0060 | 0.0093 | 0.6687 | 1.0000 | 1.0000 | 1.0000 |
| Meandering | 0.1430 | 0.1520 | 1.0000 | 1.0000 | 1.0000 | 1.0000 |

**Table S7** Y-Maze test: P-values for two-way repeated measure ANOVA followed by Bonferroni's post hoc test.

|  | Time immobile (s) | | | Total distance travelled (m) | | | Average speed (m/s) | | |
| --- | --- | --- | --- | --- | --- | --- | --- | --- | --- |
|  | 18°C | 26 °C | 34°C | 18°C | 26 °C | 34°C | 18°C | 26 °C | 34°C |
| T1 vs T2 | 1.0000 | 1.0000 | 1.0000 | 1.0000 | 0.5185 | 1.0000 | 1.0000 | 0.4793 | 1.0000 |
| T1 vs T3 | 0.8858 | 1.0000 | 1.0000 | 1.0000 | <0.05 | 1.0000 | 1.0000 | <0.01 | 1.0000 |
| T1 vs T4 | 1.0000 | 1.0000 | 1.0000 | 1.0000 | <0.001 | 1.0000 | 1.0000 | <0.001 | 1.0000 |
| T2 vs T3 | 1.0000 | 1.0000 | 1.0000 | 1.0000 | 1.0000 | 1.0000 | 1.0000 | 1.0000 | 1.0000 |
| T2 vs T4 | 1.0000 | 1.0000 | 1.0000 | 1.0000 | 1.0000 | 1.0000 | 1.0000 | 1.0000 | 1.0000 |
| T3 vs T4 | 0.4848 | 1.0000 | 1.0000 | 1.0000 | 1.0000 | 1.0000 | 1.0000 | 1.0000 | 1.0000 |

|  | Maximum speed (m/s) | | | Absolute turn angle (°) | | | Transitions (N) | | |
| --- | --- | --- | --- | --- | --- | --- | --- | --- | --- |
|  | 18°C | 26 °C | 34°C | 18°C | 26 °C | 34°C | 18°C | 26 °C | 34°C |
| T1 vs T2 | 1.0000 | 1.0000 | 1.0000 | 1.0000 | 0.9626 | 1.0000 | 1.0000 | 1.0000 | 1.0000 |
| T1 vs T3 | 1.0000 | 1.0000 | 1.0000 | 1.0000 | <0.05 | 1.0000 | 1.0000 | <0.05 | 1.0000 |
| T1 vs T4 | 1.0000 | 1.0000 | 1.0000 | 0.5488 | <0.0001 | 1.0000 | 1.0000 | <0.001 | 1.0000 |
| T2 vs T3 | 1.0000 | 1.0000 | 1.0000 | 1.0000 | 1.0000 | 1.0000 | 1.0000 | 1.0000 | 1.0000 |
| T2 vs T4 | 1.0000 | 1.0000 | 1.0000 | 0.4168 | 0.4921 | 1.0000 | 1.0000 | 1.0000 | 1.0000 |
| T3 vs T4 | 1.0000 | 1.0000 | 1.0000 | 1.0000 | 1.0000 | 1.0000 | 1.0000 | 1.0000 | 1.0000 |

|  | Time spent in N arm (%) | | | Entries in N arm (N) | | | Entries in sector 3  of the N arm (N) | | |
| --- | --- | --- | --- | --- | --- | --- | --- | --- | --- |
|  | 18°C | 26 °C | 34°C | 18°C | 26 °C | 34°C | 18°C | 26 °C | 34°C |
| T1 vs T2 | 1,0000 | 1,0000 | 1,0000 | 1,0000 | 0,9296 | 1,0000 | 1,0000 | 1,0000 | 1,0000 |
| T1 vs T3 | 1,0000 | <0,05 | 1,0000 | 1,0000 | 0,0635 | 1,0000 | 1,0000 | <0,001 | 1,0000 |
| T1 vs T4 | 1,0000 | <0,05 | 1,0000 | 1,0000 | <0,001 | 1,0000 | 1,0000 | <0,0001 | 1,0000 |
| T2 vs T3 | 1,0000 | 1,0000 | 1,0000 | 1,0000 | 1,0000 | 1,0000 | 1,0000 | 0,1361 | 1,0000 |
| T2 vs T4 | 1,0000 | 1,0000 | 1,0000 | 1,0000 | 1,0000 | 1,0000 | 1,0000 | <0,05 | 1,0000 |
| T3 vs T4 | 1,0000 | 1,0000 | 1,0000 | 1,0000 | 1,0000 | 1,0000 | 1,0000 | 1,0000 | 1,0000 |
